# Tetrodotoxin and the state-of-the-art progress of its associated analytical methods

**DOI:** 10.3389/fmicb.2024.1413741

**Published:** 2024-09-03

**Authors:** Wei Mi, Sha Liu

**Affiliations:** School of Public Health, Binzhou Medical University, Yantai, China

**Keywords:** tetrodotoxin, detection, aptasensor, immunosensor, cell biosensor

## Abstract

Tetrodotoxin (TTX), which is found in various marine organisms, including pufferfish, shellfish, shrimp, crab, marine gastropods, and gobies, is an effective marine toxin and the cause of many seafood poisoning incidents. Owing to its toxicity and threat to public health, the development of simple, rapid, and efficient analytical methods to detect TTX in various food matrices has garnered increasing interest worldwide. Herein, we reviewed the structure and properties, origin and sources, toxicity and poisoning, and relevant legislative measures of TTX. Additionally, we have mainly reviewed the state-of-the-art progress of analytical methods for TTX detection in the past five years, such as bioassays, immunoassays, instrumental analysis, and biosensors, and summarized their advantages and limitations. Furthermore, this review provides an in-depth discussion of the most advanced biosensors, including cell-based biosensors, immunosensors, and aptasensors. Overall, this study provides useful insights into the future development and wide application of biosensors for TTX detection.

## 1 Introduction

Tetrodotoxin (TTX) is a low-molecular weight marine toxin that is typically associated with fatal food poisoning (Katikou et al., 2022). TTX has been reported in various marine organisms, including pufferfish and shellfish, and other organisms, including cockroaches, salamanders, frogs, cockroaches, and flatworms; however, its source is still controversial (Ito et al., 2023). Moreover, owing to its thermal stability, TTX is not inactivated during the traditional cooking of pufferfish or contaminated seafood (Zhang et al., 2023). TTX is a powerful neurotoxin and can cause severe diseases (Liu Z. et al., 2023; Alkassar et al., 2024), such as tongue numbness, nausea, vomiting, respiratory paralysis, diarrhea, and abdominal pain, and in extreme cases, patients poisoned with TTX may die. The mortality rate of TTX poisoning is considerably greater than that of traditional marine poisoning (Lin and Hwang, 2012) (such as severe ciguateric toxin poisoning). Considering the severity of TTX poisoning, relevant regulations have been established by certain countries and organizations. To minimize the risk of poisoning, a simple, rapid, economical, and effective method is required for TTX detection in food.

**Figure 1:**
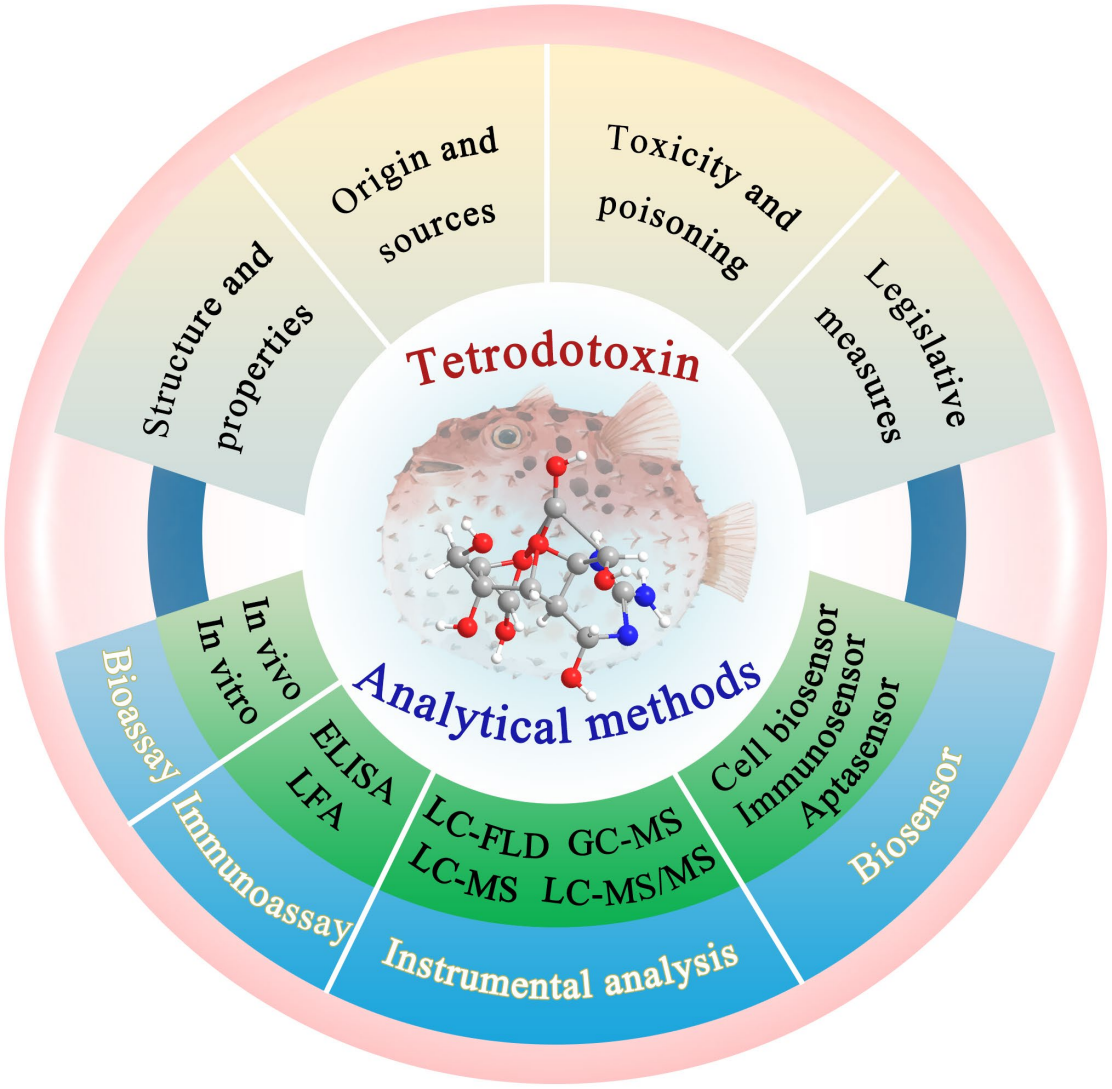
Overview of the TTX and its analytical methods. The illustration was created using BioRender.com.

Many novel technologies for TTX detection have been developed. Among them, bioassays, immunoassays, and instrumental analysis play increasingly important roles in the performance characterization and quantitative analysis of TTX (Suzuki, 2016; Hu et al., 2022; Mills et al., 2022). Additionally, biosensors have become a research hotspot in seafood analyses in recent years (Wang et al., 2022; Shati et al., 2023), and because of their unique advantages and great potential for rapid and sensitive detection of trace TTX, biosensors deserve more attention (Reverte et al., 2023). Notably, the development, advantages, and disadvantages of these TTX-detection technologies, namely, bioassays, immunoassays, instrumental analysis, and biosensors, have not been systematically summarized and comprehensively discussed. Therefore, this study mainly reviews the latest developments in these technologies over the past 5 years (Figure 1). Bioassays include mouse bioassay (MBA) and cell bioassay. Immunoassays include enzyme-linked immunosorbent assay (ELISA) and lateral flow assay (LFA). Instrumental analysis is mainly performed through liquid chromatography with fluorescence detector (LC-FLD), gas chromatography-tandem mass spectrometry (GC–MS), liquid chromatography-mass spectrometry (LC–MS), liquid chromatography coupled to tandem mass spectrometry (LC–MS/MS), high-performance liquid chromatography–tandem mass spectrometry (HPLC–MS/MS), and ultra-high performance liquid chromatography–tandem mass spectrometry (UPLC–MS/MS). Herein, we have highlighted the most advanced biosensing technologies, such as cell biosensors, immunosensors, and aptasensors. Additionally, this review provides certain key opinions and future development trends as a valuable reference for further research.

## 2 Structure and properties of TTX

TTX is a small-molecule, nonprotein marine neurotoxin with strong activity. The chemical study of TTX began in 1909. It was first discovered and isolated from wild *Tetraodontidae* by the Japanese scientist Yoshizumi Tahara (Noguchi, 2008) and named “tetrodotoxin.” Only after the 1950s did scientists successfully isolate crystalline TTX from pufferfish ovaries (Brown and Mosher, 1963). The crude product is a brown–yellow powder and the pure product is a white crystal, that is colorless and odorless. TTX exhibits slight solubility in water; it is easily soluble in acidic aqueous solutions (pH 3–7), whereas it is insoluble in organic solvents such as absolute ethanol, ether, and benzene. TTX is stable in weakly acidic aqueous solutions but easily decomposes in alkaline aqueous solutions (Hanifin, 2010); therefore, an aqueous solution of acetic acid is often used for TTX extraction. TTX remains stable in heat and has no definite melting point (Makarova et al., 2019; Zhang et al., 2023). It begins to carbonize above 220°C. Furthermore, it is not easily decomposed by enzymes such as pancreatic enzymes, salivary amylase, or emulsifying enzymes.

**Figure 2:**
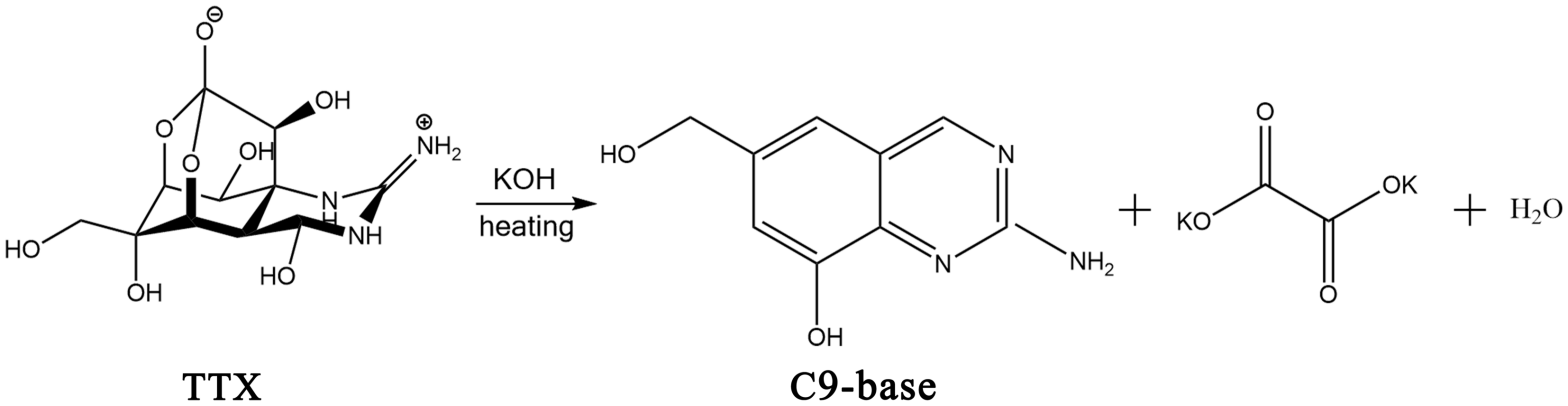
Chemical structures of TTX and its hydrolysis process in alkaline solution. The illustration was created using ChemDraw software.

In 1964, the structure of the TTX was elucidated by Robert Burns Woodward from Harvard University (Woodward and Gougoutas, 1964). In 1970, X-ray crystallography confirmed that TTX is a cage-shaped, ortho-ester alkaloid, and an amino-perhydroquinzaline compound (Lorentz et al., 2016). The molecular formula of TTX is C_11_H_17_O_8_N_3,_ and the relative molecular mass is 319.27. Although TTX has a low molecular weight, it has a very complex structure. It comprises a carbon ring, a guanidine group, and six hydroxyl groups. The chemical structure of TTX is shown in Figure 2. A separate ring is connected at the C-5 and C-10 positions by a semi-aldose lactone (Chau et al., 2011). The carbon atoms in the molecule exhibit asymmetric substitution. Additionally, TTX is a special organic compound containing both guanidine and orthoacid functional groups. Guanidine and nitrogen atoms are protonated with positive charges, and the positive carbonyl acid is dissociated into anions with negative charges. Therefore, TTX usually exists in the internal salt form (Hinman and Du Bois, 2003). Owing to its relatively stable physical and chemical properties, structural degradation of TTX by a general process is challenging. The zwitterionic structure of TTX makes it unstable under strongly acidic and alkaline conditions; hence, TTX is generally degraded using a 5% potassium hydroxide solution at 90–100°C (Figure 2). The obtained hydrolysate contains a yellow crystalline structure of 2-amino-6-hydroxymethyl-8-hydroxyquinazoline (Stan’kov et al., 2016) (referred to as the C9 base), which exhibits fluorescence characteristics and is one of the theoretical bases of chemical detection methods for TTX.

## 3 Origin and sources of TTX

The origin of TTX is still inconclusive. Presently, studies are divided into two main viewpoints: exogenous theory and endogenous theory. In 1985, Matsui et al. reported that pufferfish fed nontoxic feed after hatching through artificial cultivation of pufferfish seedlings became nontoxic; this study introduced the *in vitro* origin of pufferfish, which opened the research boom of the exogenous origin theory (Jal and Khora, 2015). This theory suggests that all TTX-producing organisms are closely related to TTX-secreting microorganisms. Next, various TTX-producing bacteria, such as Vibrio, Actinobacteria, Bacillus, and Pseudomonas, were isolated and extracted. The findings indicated that the TTX found in pufferfish was closely related to marine bacteria. In 1990, Nagashima et al. proposed the food-chain-mediated enrichment of TTX in pufferfish. The immunohistochemistry results showed that the puffer tissue sections could not secrete TTX but could absorb TTX in the culture medium, and the ability of the thin sections of the puffer liver to absorb TTX was greater than that of the thin sections of other fish livers (Gao et al., 2020). These findings confirmed the *in vitro* derivation of TTX and revealed the source of the food chain. Therefore, most researchers supporting the exogenous theory believe that TTX in organisms such as pufferfish, shellfish, and snails is mainly produced by marine microorganisms. These microorganisms are attached to the surface or body of organisms and are transmitted and enriched through the food chain, resulting in the dual influence of marine microorganisms and the food chain on TTX enrichment.

In 1995, Matsumura et al. reported that TTX extracted from *Vibrio alginalyticus* did not specifically bind to TTX monoclonal antibodies (Matsumura, 1995), which calls into question the exogenous theory that bacteria secrete TTX. Studies on the endogenous theory suggested that symbiotic microorganisms in organisms such as pufferfish may exhibit some transformation mechanism to secrete and accumulate TTX. The endogenous theory suggests that the host provides optimal growth and metabolic conditions by regulating the internal environment of its organs, and TTX is considered to be the product of the organism and the symbiotic microorganisms. Currently, various TTX-producing symbiotic microorganisms have been isolated from different species, such as pufferfish, chaetognaths, mollusks, and echinoderms. It was reported that the amount of TTX produced by pufferfish was much greater than that produced by laboratory fermentation strains. Furthermore, the mechanism underlying TTX accumulation in pufferfish can be explained by the biological enrichment through the food chain, which starts from bacteria (Noguchi, 2008). However, studies confirming whether organisms such as pufferfish can secrete TTX themselves and how TTX is transferred to various organs of their bodies are lacking.

**Figure 3:**
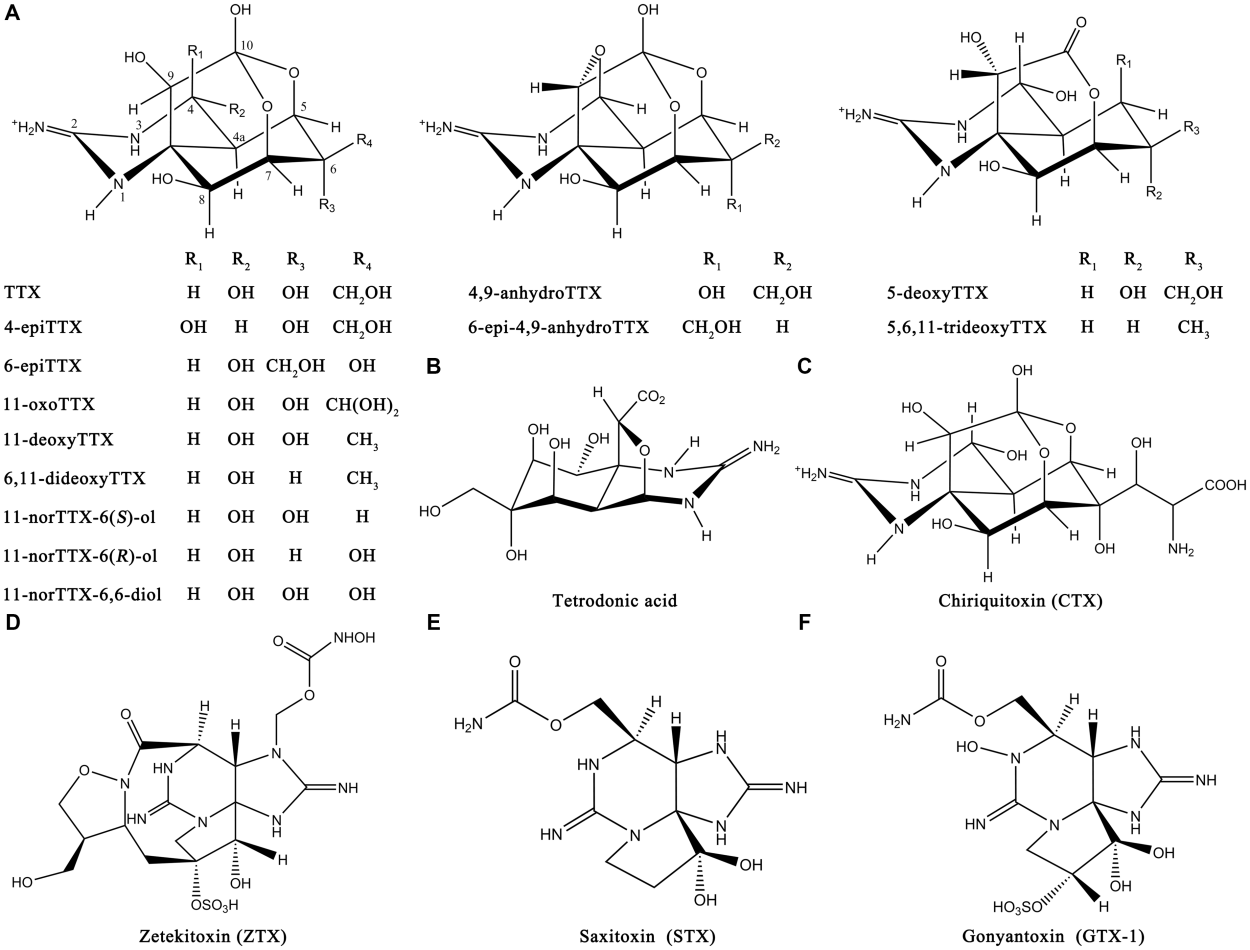
Chemical structures of TTX derivatives and structural analogs. **(A)** Common TTX derivatives. **(B)** Tetrodonic acid. **(C)** CTX. **(D)** ZTX. **(E)** STX. **(F)** GTX-1. The illustration was created using ChemDraw software.

TTX is widely found in various species and most commonly in the *Tetrodontidae* family such as pufferfish. It is also widely distributed in other vertebrates, such as shellfish, echinoderms, platyhelminth, mollusks, and some amphibians (Miyazawa and Noguchi, 2001). In addition to TTX, many TTX derivatives and structural analogs have also been isolated and identified in organisms such as snails, salamanders, flatworms, shellfish, and bacteria (Hu et al., 2022). The most common TTX derivatives include 4-epiTTX, 11-oxoTTX, 6,11-dideoxyTTX, 4,9-anhydroTTX, and 5-deoxyTTX. The most common structural analogs of TTX include tetrodonic acid, chiriquitoxin (CTX), zetekitoxin (ZTX), saxitoxin (STX), and gonyantoxin-1 (GTX-1). The chemical structures of some derivatives and structural analogs are shown in Figure 3. These compounds also exhibit certain toxicity and can cause food poisoning.

The distribution of TTX in pufferfish shows regional, seasonal, and individual characteristics. Poisoned pufferfish have been reported to be distributed in many regions (Lago et al., 2015; Guardone et al., 2019), such as Europe, Asia, and the Pacific. The development and spawning periods of pufferfish generally occur during the spring and summer, respectively, every year. During these periods, the ovarian TTX production capacity increases, whereas the liver TTX production fluctuates. Generally, the TTX distribution in pufferfish from high to low is as follows: ovary, spleen, liver, blood, gill, skin, and testis. The muscles of pufferfish are less toxic *in vivo*, but after the death of the fish or improper human treatment, TTX from highly toxic parts, such as the ovary and liver, can spread to the muscle, causing increased toxicity. Additionally, the TTX content in closely related pufferfish has been reported to be similar; however, owing to the influence of geographical location, species, and season, the TTX distribution in the organs and tissues of pufferfish differs (Ito et al., 2022).

## 4 Mechanism of action of TTX

**Figure 4:**
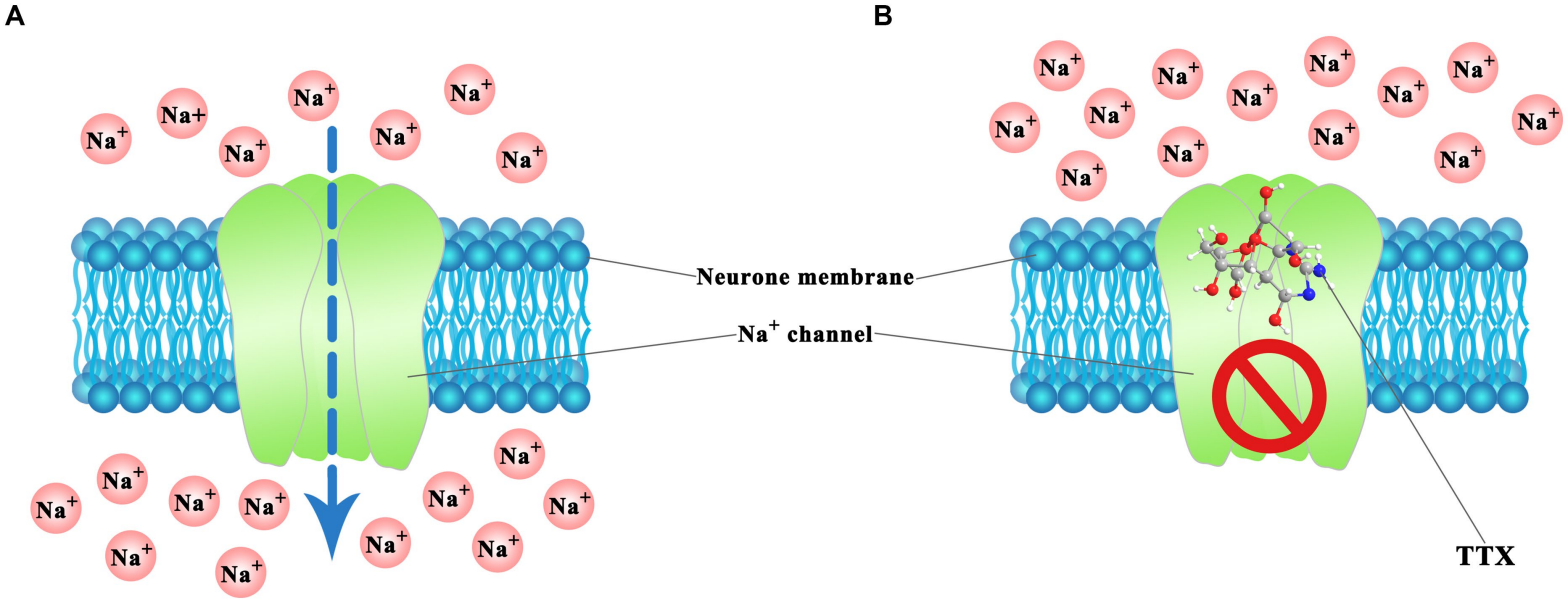
TTX action mechanism in voltage-gated Na^+^ channels of neuron cell. **(A)** Normal ion passing. **(B)** Blocked by TTX. The illustration was created using BioRender.com.

The mechanism of action of TTX has been well studied, and its toxicological effect is mainly characterized by its ability to inhibit nerve and muscle conduction. The TTX molecule, endowed with its distinctive chemical architecture, effectively blocks the passing of sodium ions through the cell membranes of nerve cells. TTX acts as a sodium channel blocker and selectively acts on sites of voltage-gated sodium channels (Figure 4) (Guillotin and Delcourt, 2021). Voltage-gated sodium channels constitute pivotal ion conduits that underpin the resting potential and neuronal excitability, both indispensable processes for the initiation and dissemination of action potentials within neurons. The structure–activity relationship of TTX indicates that the active groups of TTX are the guanidine amino groups at positions 1, 2, and 3 and the hydroxyl groups at positions C-4, C-9, and C-10. Among these, the guanidine group plays a crucial role in blocking sodium channels (Geffeney and Ruben, 2006). At physiological pH, TTX forms a positively charged active region by protonation and specifically interacts with the negatively charged carbonyl group of the sodium ion (Na^+^) channel receptor protein through hydrogen bonds. Blocking the Na^+^ channel on the nerve excitation membrane inhibits the depolarization and conduction of action potentials in nerve and muscle cells, resulting in nerve and muscle paralysis, respectively. The severity of TTX-induced symptoms depends on the dose (Homaira et al., 2010). Mild symptoms include perioral tingling, headache, sweating, muscle weakness, nausea, vomiting, abdominal pain, and diarrhea. Severe symptoms include hypotension, arrhythmia, muscle paralysis, limited movement, ataxia, and death due to respiratory failure (Bane et al., 2014; Lorentz et al., 2016; Knutsen et al., 2017).

## 5 Toxicity and poisoning of TTX

TTX is a nonprotein neurotoxin and one of the most toxic, natural high-efficiency toxins. Its toxicity is >1,250 times greater than that of sodium cyanide (Bane et al., 2014). The lethal potency of TTX ranges from 5,000 to 6,000 MU/mg, where 1 MU (mouse unit) is defined as the quantity of toxin necessary to cause mortality in a 20-gram male mouse within 30 min post-intraperitoneal injection. Furthermore, the estimated minimum lethal dose (MLD) for humans is approximately 10,000 MU, equivalent to 2 mg of TTX (Noguchi and Ebesu, 2001). However, this value may exhibit variability contingent upon the age and health status of the victims. The toxicity of TTX has been conducted in different experimental animals (including mice, rats, and rabbits) to determine some toxicity parameters. For mice, the median lethal dose (LD_50_) was 232 μg/kg for oral administration (Abal et al., 2017). On the other hand, the LD_50_ values obtained in mice were 532, 12.5, and 10.7 μg/kg for intragastric, subcutaneous, and intraperitoneal administrations, respectively (Lago et al., 2015). For rabbits, the lethal dose (LD_100_) values were 3.8 and 5.8 μg/kg, and the MLD values of TTX were 3.1 and 5.3 μg/kg, for intravenous and intramuscular administrations, respectively (Katikou et al., 2022). For rats, LD_50_ of TTX was 517.43 μg/kg for oral administration (Hong et al., 2018). Therefore, although the activity values of TTX toxicity were variation depending on experimental models and different animals, these observations have demonstrated the highly toxicity of TTX across all mammalian species.

On the other hand, TTX can be used as a tool drug in neurophysiology, pharmacology, and other research fields owing to its unique properties, such as high specificity to voltage-dependent Na^+^ channels, selective blocking of Na^+^ channels, and inability to cross the blood–brain barrier. An appropriate dose of TTX can rapidly and effectively relieve pain in patients, playing a major role in sedation, analgesia, and local anesthesia (Zhao et al., 2019; Konrad et al., 2022). In addition to its use as an anesthetic and analgesic, TTX can also be used as a withdrawal and anticancer drug (Shi et al., 2009; Yildirim et al., 2012), which has potential medical development value. However, in the fields concerning food safety and military operations, TTX can be dangerous to human life, and its toxicity cannot be ignored.

TTX is widely present in various seafoods, such as pufferfish, shellfish, and snails, and its high toxicity is an important factor in many food poisoning incidents. A recent retrospective study of human TTX poisoning cases caused by global consumption of seafood (Guardone et al., 2019) reported that as of April 2018, among the 3,032 cases from five continents, Asia presented the largest number of cases (88.6%), followed by Africa, America, Oceania, and Europe. Among the TTX seafood poisoning incidents, the main source of poisoning was fish (59.9%), followed by gastropods, arthropods, and cephalopods. The most toxic fish was pufferfish. Pufferfish are tight, delicious, and nutritious; therefore, they are highly consumed in Asia, such as in Japan, southeast coastal areas of China, South Korea, and Vietnam, where poisoning incidents caused by accidental consumption of toxic pufferfish occur frequently. In Asia, mortality due to TTX-related food poisoning is reported to be as high as 10.4%.

The toxic dose of TTX is low, the incubation period is short, and the mortality rate is high. Currently, there is no specific antidote for TTX (Wang et al., 2023b). If the rescue is not timely, in severe cases, the patient may die within 20 min, which seriously affects the quality and safety of aquatic products and human health (Morabito et al., 2017). Despite the safety risks associated with the consumption of TTX-contaminated pufferfish, their delicious taste, rich nutritional status, and high economic value often make the repeated consumption of wild pufferfish difficult. Furthermore, during processing, the highly toxic parts of pufferfish are often discarded as waste, which also poses certain potential hazards. This signifies the establishment of TTX-related policies and regulations (Liu Z. et al., 2023).

## 6 Relevant legislative measures

To effectively control TTX poisoning incidence and ensure the safety of public diets, many countries have established relevant legislative measures. Japan stipulates that the acceptable threshold of TTX in the edible parts of pufferfish is 10 MU/g, which is equivalent to the regulatory limit of TTX in 2 mg/kg pufferfish tissues (Katikou, 2019). Although there is no clear limit stated by the United States (U.S.), imported pufferfish are strictly regulated by the U.S. Food and Drug Administration (Cohen et al., 2009). TTX cannot be detected in any product sold legally (Yakes et al., 2010). Although the European Union (EU) has not set the maximum allowable content of TTX in seafood, the current legislation stipulates that pufferfish or associated fishery products cannot enter the European market (Lago et al., 2015). Some non-EU Mediterranean countries (Kosker et al., 2019), such as Turkey and Egypt, also implemented similar regulatory requirements. In 2017, at the request of the European Commission, the European Food Safety Authority issued an opinion on the public health risks of TTX in marine bivalves and gastropods (Knutsen et al., 2017). It has been proposed that a concentration of <44 μg/kg TTX in shellfish meat will not adversely affect the human body. LC–MS/MS is the most suitable method for detecting TTX and its analogs, and the limit of quantification is 0.1–25 μg/kg. In China, the Ministry of Agriculture and the Food and Drug Administration prohibited the operation of live-farmed pufferfish, unprocessed whole pufferfish, and all varieties of wild pufferfish, and clearly stated that the TTX content of pufferfish products should not exceed 2.2 mg/kg (fresh).

To further strengthen the authority of quality supervision of aquatic products such as pufferfish, strengthening the whole process monitoring of aquatic product circulation and improving accurate quantitative detection technology for TTX in food are necessary. Therefore, high-performance detection and analysis methods for broader application prospects and the efficient detection of hazardous substances are urgently needed.

## 7 Analytical methods for TTX

TTX is mainly detected through bioassays, immunoassays, instrumental analysis methods, and biosensors. These methods exhibit a dual nature, encompassing both advantageous features and inherent limitations. The MBA was the first test for detecting TTX. The results were determined by analyzing the acute effects of intraperitoneal TTX injection on animals. However, its low sensitivity and accuracy and associated animal ethical problems limit its wide application. Next, various immunoassays and instrumental analysis methods have been developed that eliminate animal ethical issues and have made substantial progress in recent years. Furthermore, studies on developing more rapid, sensitive, and accurate biosensor methods for accurate quantitative analysis of TTX in different matrices are being conducted. Herein, the development and application of four analytical methods for TTX detection are discussed.

### 7.1 Bioassays

The bioassays for TTX detection are mainly divided into *in vivo* and *in vitro* bioassays.

#### 7.1.1 *In vivo* bioassay

MBA is an example of an *in vivo* analysis. The MBA method is the first TTX detection method proposed by Japan and the first standard method for detecting marine biotoxins implemented in various countries. In MBA, different concentrations of TTX are injected intraperitoneally into mice of a certain weight. The TTX concentration is then determined based on the quantitative relationship between the time of death and the TTX concentration injected into the mice. The toxicity intensity is expressed in mouse unit (MU). The Japanese authorities defined 1 MU as the dose of TTX that can induce death in a 20 g ddY male mouse within 30 min, and 1 MU is equivalent to 0.22 μg TTX (Yasumoto et al., 1995). The intuitive experimental phenomenon and lack of requirements for expensive instrumentation are the advantages of the MBA. However, this analysis method is greatly affected by various factors and individual differences in mice (Suzuki, 2016), resulting in poor accuracy and repeatability of the test results. Furthermore, ethical problems associated with laboratory animals are also involved (Campbell et al., 2011). Raising animals requires high labor, material, and financial resources. Hence, MBA is mostly used to extract toxicological information about TTX (Asakawa et al., 2019), and the test results are generally used as a reference (Kosker et al., 2016).

#### 7.1.2 *In vitro* bioassay

*In vitro* bioassays include the cytotoxicity analysis. In this method, nerve cells are cultured *in vitro* and treated with a certain concentration of veratridine and ouabain. Both induce high Na^+^ influx, causing cell swelling and necrosis, while the combination of TTX and the Na^+^ receptor blocks Na^+^ influx and repairs cell morphology. The morphology of the recovering cell is observed by microscopy. The TTX content is estimated based on the relationship between the TTX concentration and the cell recovery rate. Gallacher and Birkbeck (1992) used this method to measure the limit of detection (LOD) to be 20 ng/mL. This method presents no ethical problems, and its operation is relatively simple and more sensitive than that of the MBA. However, this method requires a long cell culture duration and has high requirements for operation steps and the experimental environment. The experimental results can be easily confused and greatly affected by subjective judgment (Guzmán et al., 2007). Therefore, it is difficult to widely apply this method in practical applications.

### 7.2 Immunoassays

Immunoassays are based on the principle of specific binding reactions between antigens and antibodies. The small molecular weight and lack of immunogenicity of TTX make the preparation of specific antibodies challenging. Reportedly, carrier proteins have been coupled with TTX to prepare artificial complete antigens, which, after immunization, induce polyclonal antibody production. Monoclonal antibodies have also been prepared using hybridoma technology. These antibodies are used in various immunoassays, such as ELISA, LFA, and other novel immune methods. These immunoassays exhibit high specificity, do not require complex or expensive instruments and are simple and fast to perform. These methods can be used for the preliminary screening of a large number of samples and can meet the needs of on-site detection. Hence, numerous ELISA detection kits have been developed by various institutions and companies. Currently, the research and development technology and processing technology are relatively mature, have achieved mass production, and are widely used in commercial applications (Zhong et al., 2011). However, the sensitivity of conventional ELISA and LFA is relatively low, and the antibody preparation process is cumbersome and costly. Moreover, the stability and repeatability of antibodies prepared in different batches can be different. Consequently, this method is mainly used for semiquantitative detection. It may be combined with other technologies for accurate quantification.

#### 7.2.1 ELISA

Based on the binding modes of the immune bodies, ELISA can be divided into direct, competitive, indirect, double antibody sandwich, and other analysis methods (Saeed et al., 2017). Vlasenko et al. (2020) developed a competitive ELISA method using a polyclonal antibody. The LOD and limit of quantitation (LOQ) of TTX were 25.54 and 777.34 ng/mL, respectively. The detection range was 25.54–2,500 ng/mL. This method successfully determined the TTX content in marine banded worms, which was further confirmed by HPLC–MS/MS, expanding the application of ELISA in invertebrates. Sato et al. (2019) obtained the TTX hapten by combining the derivatives formed by reacting 4,9-anhydro-TTX and 1,2-ethanedithiol with keyhole limpet hemocyanin. A novel polyclonal antibody against TTX was prepared after immunizing rabbits. Using biotin-labeled TTX, a direct “one-step” ELISA was developed. The LOD and detection range were 3 nM and 10–300 nM, respectively, and TTX was successfully detected in the toxic pufferfish. Notably, the different groups of hapten and different chemical synthesis methods crucially affect the cross-reactivity of different marine biotoxins. Hence, these issues should be carefully considered when interpreting the results of immunochemical assays (Magarlamov et al., 2017).

To avoid potential TTX–carrier protein coupling-induced problems, Reverté et al. (2015) proposed a strategy involving orderly directional immobilization of the TTX antigen and established a novel competitive maleimide-based ELISA (mELISA) method. In this method, dithiol is self-assembled on a maleimide plate to provide a stable, regular, and spaced monolayer, facilitating directional immobilization of antigens, improving the affinity of antigens to antibodies, and reducing the nonspecific bindings. The LOD was 2.28 ng/mL. The TTX content in the pufferfish samples was quantitatively determined using mELISA and a previously established surface plasmon resonance (SPR) immunosensor. The LC–MS/MS results were used for comparison and showed good correlation. Additionally, this method was used to analyze the cross-reactivity factors (CRFs) of the monoclonal antibody and various TTX derivatives. The CRFs measured by mELISA and SPR were different, indicating that the immobilization process of TTX in the immunoassay method was crucial. Although it is the key to improving the detection performance, the immobilization process is cumbersome and can increase the analysis time and cost. Additionally, the mELISA method was successfully applied for quantitatively analyzing pufferfish samples from Greece (Reverté et al., 2015) and Spain (Rambla-Alegre et al., 2018), along with shellfish (Reverté et al., 2018) and urine (Rambla-Alegre et al., 2018) samples.

#### 7.2.2 LFA

The antibody-based LFA is a widely used immunoassay method to detect TTX. Antibody-labeled gold nanoparticles (AuNPs) are usually used as probes; hence, they are also known as colloidal gold test strips. The results of this method require eye-based interpretation, making it a semiquantitative analysis method. The detection process is simple and fast, and it does not require special instruments, which facilitates its use as an on-site rapid detection method, making it suitable for the initial screening of large quantities of samples. Zhou et al. (2010) established a colloidal gold test strip method for the rapid detection of TTX in pufferfish tissues. The detection range was 40–8,000 ng/mL. The visual LOD of TTX-spiked samples was 40 ng/mL, and the determination time was <10 min. After purifying the anti-TTX monoclonal antibody, competitive ELISA and colloidal gold test strip methods were established by Ling et al. (2015). The linear detection range of the ELISA was 5–500 ng/mL, and the LOD was 4.44 ng/mL. The colloidal gold test strip achieved semiquantitative detection of TTX within 10 min without using any instrument. The LOD was 20 ng/mL, and spiked samples such as saury and clam were analyzed. This method has a faster response and simpler operation. Additionally, a colloidal gold test strip for the simultaneous detection of TTX and okadaic acid (OA) in seafood samples has been developed (Ling et al., 2019). Using this method, the visual detection of TTX and OA was completed within 10 min, and the limit of detection values (LODs) were 15 and 0.75 ng/mL, respectively. Furthermore, the assay was successfully used for the visual detection of clams and carp samples (Li et al., 2020).

**Figure 5:**
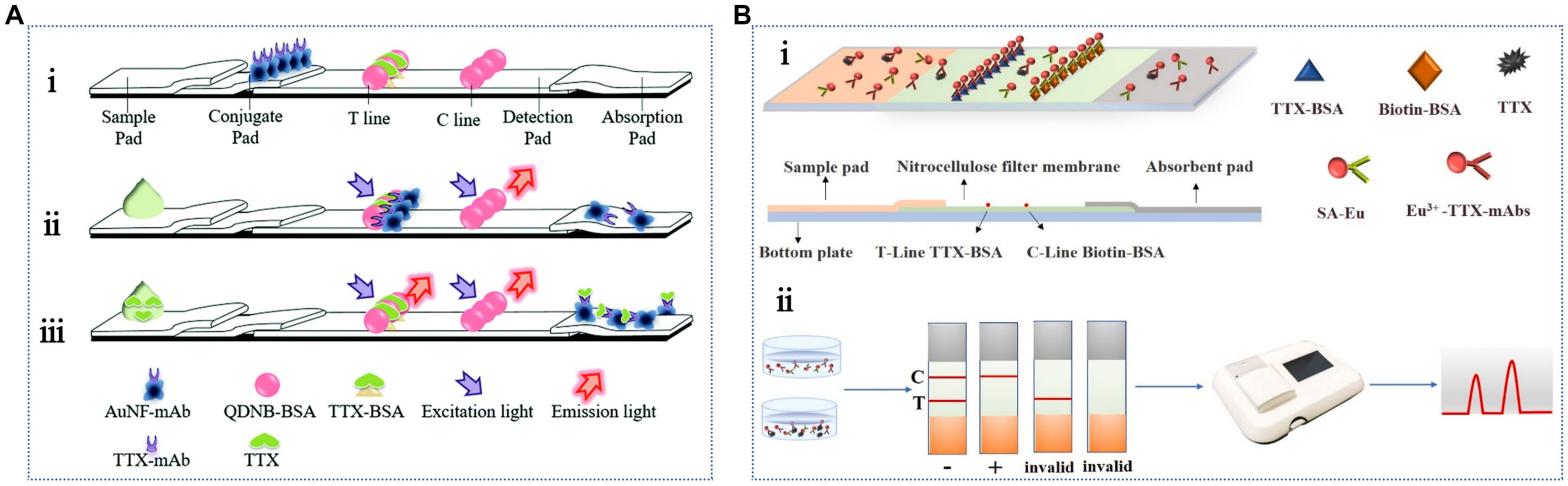
Antibody-based fluorescent LFA methods. **(A)** Schematic illustration of the competitive LFA combining QDNBs and AuNFs: (i) the structure, (ii) negative reaction, (iii) positive reaction. The illustration has been adapted and modified with permission acquired from Shen et al. (2017a). **(B)** Schematic representation of the LFA based on time-resolved fluorescent microspheres: (i) TTX detection, (ii) qualitative and quantitative test results. The illustration has been adapted and modified with permission acquired from Sun J. et al. (2023).

To avoid issues associated with subjective results, the LFA may be combined with portable readers, such as fluorescence readers, to provide more accurate measurement data. Shen et al. (2017b) developed a membrane-based fluorescence quenching immunochromatographic assay in which the fluorescent microsphere-labeled TTX-bovine serum albumin (BSA) complex and monoclonal antibody-labeled AuNPs were used as a fluorescence indicator signal and a fluorescence quencher, respectively. This assay detected TTX within 12 min, presenting a linear range of 3.13–50 ng/mL and an LOD of 0.78 ng/mL. The sensitivity of this method was improved by an order of magnitude compared with that of traditional colloidal gold test strips, as verified using TTX-spiked pufferfish samples. Although the recovery rate ranged from 61.3 to 70.4%, the practical application performance was slightly worse. Additionally, based on the fluorescence quenching effect (Figure 5A), competitive LFA methods combining quantum dot nanobeads (QDNBs) and gold nanoflowers (AuNFs) have been developed to improve analytical performance (Shen et al., 2017a). The detection range and LOD were 1.56–100 ng/mL and 0.2 ng/mL, respectively. The recovery range of the pufferfish samples was 85.5–119.7%. Compared with the previous method, the fluorescence signal intensity measured during the detection process was high, and the background interference was small, suggesting that this method is faster, more sensitive, and more practical. Sun J. et al. (2023) developed a fluorescent immune test strip using time-resolved fluorescent microspheres and conjugated anti-TTX antibodies as fluorescent labels and detection probes, respectively. As shown in Figure 5B, a streptavidin–biotin system was introduced as an independent control system to further stabilize the fluorescence intensity of the control line. The results showed rapid TTX detection within 20 min, with an LOD of 0.05 ng/mL and a linear range of 0.5–40 ng/mL. The acceptable recovery rate was 97–102%, showing the reliability and stability of the method in the shellfish samples. Additionally, AuNFs and latex microsphere-based strips have been used to successfully detect TTX in yellow croaker, grass carp, perch, and pufferfish (Huang et al., 2023).

### 7.3 Instrumental analysis

Instrumental analysis methods for TTX detection include LC-FLD, GC–MS, LC–MS, LC–MS/MS, HPLC–MS/MS, and UPLC–MS/MS.

#### 7.3.1 LC-FLD

LC-FLD is the earliest instrumental TTX detection method. TTX does not exhibit fluorescence; however, its C9 base reaction product exhibits fluorescence characteristics under heating and alkaline conditions, and it can be used for the quantitative detection of TTX. After sample purification by an immunoaffinity column, the TTX concentration is determined using post-column derivatization LC-FLD. The method is qualitative, based on retention time analysis, and quantitative, based on the external standard method. Furthermore, it has been successfully applied for TTX analysis in toad (Mebs et al., 1995) and salamander (Yotsu-Yamashita et al., 2012) samples. LC-FLD is more sensitive than the MBA. Because TTX does not show fluorescence absorption, it must be derivatized into a substance with fluorescence properties; therefore, a derivative device is mandatory, and the operation is cumbersome. Moreover, the results of this method can easily be interfered with by fluorescent impurities and are very sensitive to background fluorescence and quenching effects in the environment, which necessitates high requirements for the sample matrix. For this method, it is necessary to confirm the effects of interference factors on TTX determination and elimination of these factors.

#### 7.3.2 GC–MS

GC–MS utilizes gases and volatile organic compounds for compound detection. TTX is a heat-stable, nonvolatile compound; therefore, it needs to be detected after derivatization. Man et al. (2010) used acetic acid to extract TTX from various pufferfish samples. After derivatization with N-methyl-N-(trimethylsilyl) trifluoroacetamide at 60°C for 30 min, its derivative was subjected to GC–MS-mediated detection. The LOD was 0.5 μg/g, and the detection time was 8.2 min. The solvent and derivatization agent contents in the pretreatment samples were low, which met the requirements of routine TTX detection. Stan’kov et al. (2016) used a similar method to determine the TTX content in water, plasma, and drug samples (0.05–1.0 μg/mL). However, neither GC–MS nor LC-FLD can distinguish among the structural analogs of TTX, and both methods require derivatization. Therefore, in recent studies, these two methods have been replaced by other MS-based detection techniques. MS-based methods provide higher specificity and improve the detection of some TTX analogs with low signal intensity (Shoji et al., 2001).

#### 7.3.3 LC–MS and LC–MS/MS

LC–MS and LC–MS/MS (including HPLC–MS/MS and UPLC–MS/MS) are the most commonly used instrumental TTX detection methods. Following extraction, the samples are purified using immunoaffinity columns, and the mixture is separated through LC. The separated components are then detected through MS, which simultaneously performs qualitative and quantitative analyses. The isolation and identification abilities of these methods are efficient and rapid, with high sensitivity and accuracy, and they can achieve high-throughput and trace analysis. The analysis results are authoritative, and these methods are widely applicable to various samples. Derivatization is not required for these methods, which greatly simplifies the analysis workload. Horie et al. (2002) established an LC–MS method for TTX detection via electrospray ionization in selected ion monitoring (SIM) mode. The linear range was 0.01–1 mg/mL. The method was applied to analyze spiked pufferfish samples, and a recovery rate of 77.7–80.7% and an LOD of 0.1 mg/kg were obtained. Because the structures of TTX and its analogs are very similar, their relative response coefficients in the SIM mode of LC–MS are similar; therefore, TTX is also used as an internal standard when quantifying TTX analogs (Chen et al., 2011).

To improve the isolation and enrichment of TTX, the Yotsu-Yamashita group (Nakagawa et al., 2006; Yotsu-Yamashita et al., 2010) combined hydrophilic interaction liquid chromatography (HILIC) columns with MS to develop a HILIC-MS/MS method. Compared with that of LC–MS, the LOD of HILIC-MS/MS was three times lower, and it could be successfully applied to the quantitative analysis of pufferfish (Jang and Yotsu-Yamashita, 2006; Jang et al., 2010) and gastropods (Jen et al., 2014). Additionally, multiple reaction monitoring methods have also been applied for accurate quantitative analysis of bivalves (Leão et al., 2018; Watanabe et al., 2019). To solve the problem of large sample volumes in HILIC-MS/MS, Long et al. (2020) developed a novel pulse diffusion focusing (PDF)-based sampling technique. The PDF-HILIC-MS/MS method only requires a milliliter-level sample volume, which suggests that it is more sensitive than traditional sampling methods. Furthermore, this approach solved the challenges of offline sample preparation and solvent incompatibility in HILIC by developing an automatic analysis system. The system was used for determining TTX in the plasma and urine samples, and the detection ranges were 2.2–400 and 7.3–400 ng/mL, respectively, while the LODs were 0.65 and 2.2 ng/mL, respectively. This method is universal for different hydrophilic toxins.

Additionally, LC–MS/MS, HPLC–MS/MS, and UPLC–MS/MS are widely used for analyzing TTX. Knutsen et al. (2017) used this method to determine the TTX content in marine gastropods and bivalves in European waters, with LODs ranging from 0.1–25 μg/kg. Another LC–MS/MS method was developed for determining TTX and its analogs in high- and low-fat tissues of pufferfish, and its application was validated in Korean pufferfish (Park et al., 2024). Chen et al. (2017) developed a solid-phase microextraction fiber for extracting TTX from spiked pufferfish samples, which showed better extraction performance than that of commercial materials. Subsequently, LC–MS/MS was used for TTX analysis. The linear range was 10–1,000 ng/g with an LOD of 2.3 ng/g. The detection sensitivity of this method was lower than the national standard. The quantitative detection of TTX was performed in the dorsal muscle of living pufferfish. Ye et al. (2022) established an HPLC–MS/MS method, which presented the advantages of a wide detection range (0.2–100 ng/g), low detection limit (0.2 ng/g), and high accuracy (recovery rate of 90.5–107.2%). This method has also been successfully applied for determining TTX in various fresh and hot-processed aquatic products. Nzoughet et al. (2013) employed two different sample preparation techniques (namely, accelerated solvent extraction and simple solvent extraction) combined with UPLC-MS/MS to quantitatively analyze TTX in conch and pufferfish samples. The LOD was 1.46 ng/mL, indicating good recovery (80–92%). Meng et al. (2022) coupled solid-phase microextraction with UPLC-MS/MS for *in vivo* detection of TTX in *Takifugu obscurus*. The LOD and LOQ were 32 and 150 ng/g, respectively. Compared with LC-MS, UPLC-MS/MS presented a shorter separation time, higher separation efficiency, and higher resolution. In addition, the HILIC technique is increasingly used for chromatographic separation of TTX with LC-MS/MS, because it can effectively separate polar substances without the use of ion pair reagents. Using HILIC with internal standard calibration, Qian et al. (2024) detected TTX analogs in bivalve mollusks and monitored and evaluated TTX contamination in bivalve mollusks collected from markets in Zhejiang Province, China.

In practical applications, TTX often coexists with many other toxins, which has accelerated the development of instrumental analysis methods for detecting various marine biotoxins (Boundy et al., 2015; Rodríguez et al., 2018; Patria et al., 2020; Turner et al., 2020). Rey et al. (2018) used a porous graphite carbon column with an LC-MS/MS method to detect paralytic shellfish toxins (PSTs) and TTX and successfully analyzed 13 hydrophilic PSTs and TTX and 6 hydrophobic PSTs within 20 min. Ochi (2021) combined ion-pair solid phase extraction technology and HILIC-MS/MS to simultaneously determine the contents of 10 PSTs and TTX in scallops and clams. The average recoveries of these 11 analytes were 75.7–96.2%, and the LODs of TTX were 27.4–27.9 μg/kg. Another new analytical method combining modified QuEChERS (Quick, Easy, Cheap, Effective, Rugged, Safe) with LC tandem Q-Exactive Orbitrap high-resolution MS (LC/Q-Orbitrap-HR-MS) was reported for the simultaneous analysis of 10 PSTs and 2 TTXs in human serum (Zheng et al., 2023). The average recoveries of all toxins were 85.3–118.2%. Furthermore, this method was applied to real human serum samples obtained from intoxication cases caused by consumption of toxin-contaminated Gastropoda (*Bullacta exerata*), showing its potential for accurate diagnosis of toxin-poisoning patients in the clinic. Notably, the TTX detected by the LC-MS/MS method exhibited a low LOD, high recovery rate, and good resolution. This method presents the complementary advantages of chromatography and MS, and it is suitable for TTX detection and trace analysis. However, it has high requirements for sample purity, including other challenges of a complex detection process, such as high analysis cost and expensive precision instruments.

### 7.4 Biosensors

Biosensors use specific biomolecules as recognition elements. They convert the signals recognized by biomolecules into measurable electrical or chemical signals via transducers. This method possesses the advantages of high sensitivity, strong specificity, simple operation, diversified functions, and ease of miniaturization. Hence, it has garnered more attention in TTX detection. Cells, antibodies, and aptamers are common biometric elements used in TTX sensing. TTX biosensors can be divided into three main types: cell biosensors, immunosensors, and aptasensors.

#### 7.4.1 Cell biosensor

Cell biosensors use living cells as sensitive elements, producing different types of signal changes after sensing stimulation, including cell metabolism, action potentials, and electrical impedance. The transducer can detect changes in signals and convert them into measurable electrical signals. Because TTX is highly specific to the nervous and cardiovascular systems, the cell-sensitive components are primarily neurons and cardiomyocytes. These two cells are electrically excited, and their cell membranes are highly expressed. Na^+^ channels can produce action potentials. The two types of cells also differ in their sensitivity to TTX. Heinemann et al. (2012) reported that neuronal sodium channels were the most sensitive to TTX. Nicolas et al. (2014) cultured rat cortical neurons on a commercial 48-well plate. They used a multielectrode sensing array to detect the effects of neurotoxic model compounds, such as pure marine neurotoxins and contaminated seafood extracts, on neuronal activity. The results revealed that the sensitivity of this method was 88%. Compared with alternative *in vitro* methods, this method exhibits good repeatability and can respond to several marine biotoxins, including TTX, pacific ciguatoxin-1 (P-CTX-1), STX, PbTX-3 (brevetoxin-3), palytoxin (PlTX), and domoic acid (DA). The LOD of TTX is approximately 0.3 ng/mL. Although these toxins have different mechanisms of action, this method can determine the local field potential of cells. With the purpose of advancing the development of cell biosensors for TTX, Neuro-2a cells, as a proof of concept, were applied to the analysis of fish extracts containing TTX (*Lagocephalus sceleratus*) (Alkassar et al., 2022). Afterward, a Neuro-2a cell-based automated patch clamp (APC) system was used to detect the TTX content in pufferfish samples (Campàs et al., 2024), with an LOD of 0.05 mg TTX equiv./kg. It was also applied to the analysis of extracts of a *Lagocephalus sceleratus* specimen, providing an *in vitro* toxicological approach.

**Figure 6:**
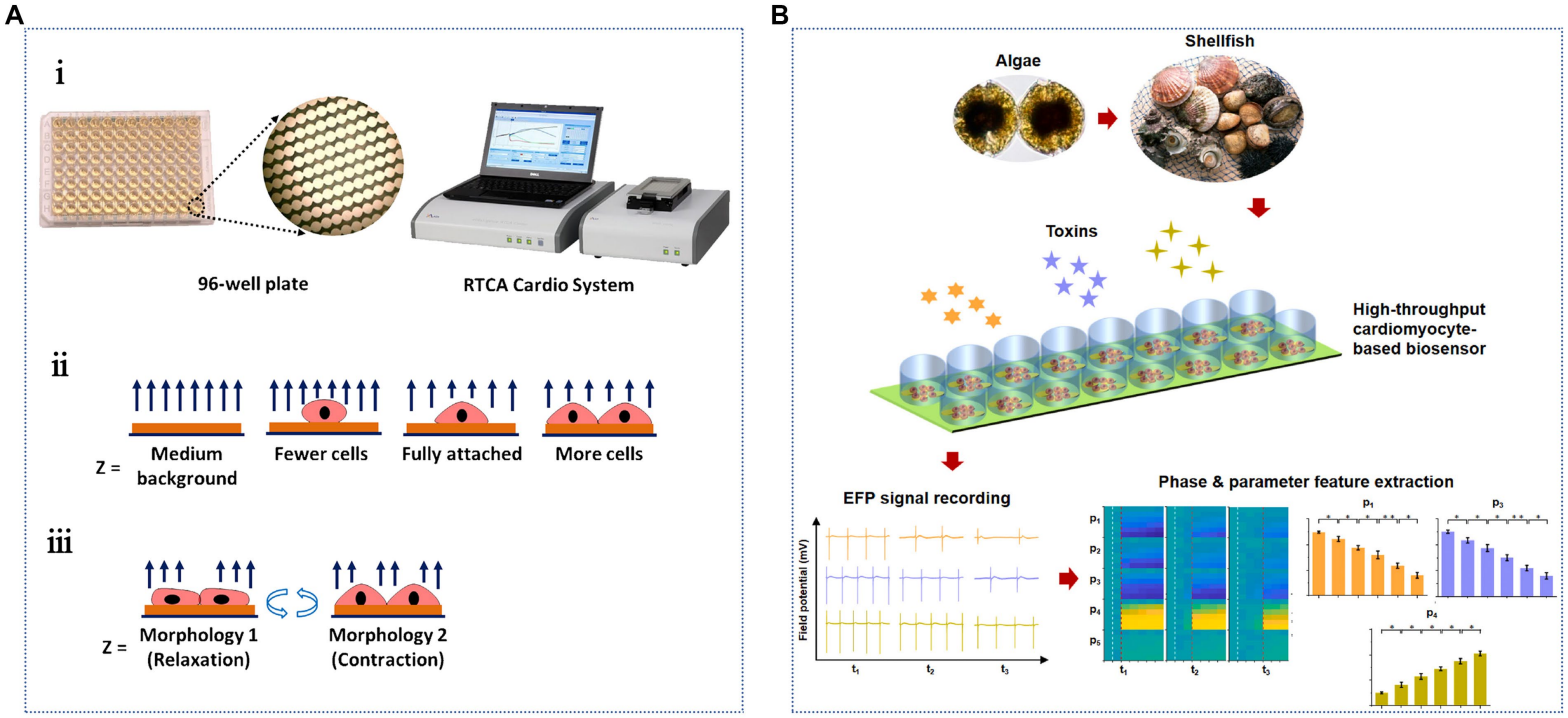
Cardiomyocyte-based TTX biosensors. **(A)** Schematic illustration of the cardiomyocyte-based impedance biosensor: (i) the 96-well sensor plate and the sensing system, impedance detection principles of (ii) cell growth and (iii) cardiomyocyte beating status. The illustration has been adapted and modified with permission acquired from Wang et al. (2015). **(B)** Schematic illustration of the specific detection of marine toxins by a high-throughput cardiomyocyte-based potential biosensor. The illustration has been adapted and modified with permission acquired from Sun X. et al. (2023).

Cardiomyocytes exhibit a unique excitation-contraction coupling mechanism, which can be used to determine the electrical excitation process of cells. Additionally, *in vitro* culturing of neurons is easier because the culture period is short, and they can grow and mature in 2–3 days. Wang et al. (2015) used a rat cardiomyocyte-based impedance biosensor to determine the pharmacological effects of STX and TTX (Figure 6A). The biosensor contained a 96-well microplate with gold microelectrodes integrated at the bottom of each well. The growth and pulsation of cardiomyocytes were monitored using a porous impedance sensor containing an interdigital electrode structure and a real-time cell analysis (RTCA) system. The impedance value correlated with the number of cells in the hole and the adhesion strength. Cell growth was quantitatively monitored based on the functional relationship between the cell index and the impedance. The LODs of STX and TTX were 0.087 and 89 ng/mL, respectively; however, the detection sensitivity of TTX was slightly lower. Notably, pulsatile signals are rhythmic changes in cell attachment and morphology due to the contraction and relaxation of cardiomyocytes, which regulate impedance signals accordingly. This method is highly sensitive, noninvasive, and allows real-time monitoring. However, this method has not been used in actual samples to detect the analytical performance of TTX, which warrants further study. They constructed a portable high-throughput cardiomyocyte-based potential biosensor containing cardiomyocytes, a 16-well microelectrodes (MEs) sensor, and a robust 32-channel recording system (Figure 6B). This biosensor can rapidly detect 0.30 ng/mL TTX within 10 min and is a promising tool for the efficient detection of ion channel toxins (Sun X. et al., 2023).

Cell biosensors enable high-throughput, nondestructive, real-time, and dynamic monitoring and analysis. Nevertheless, this method requires considerable time to culture cells before administration. Furthermore, the cell response time is long. Therefore, the procedure is cumbersome and time-consuming, and the sensitivity is low. Hence, this method is not suitable for rapid and accurate quantitative analysis and is mostly used for toxicology and pharmacological studies on TTX.

#### 7.4.2 Immunosensor

Immunosensors are based on the specific immune reaction between antibodies and antigens. These sensors mainly use antibodies as recognition elements. Currently, the developed electrochemical, optical, and other immunosensors have been successfully used to detect TTX (Tang et al., 2023). Furthermore, electrochemical immunosensors are the most commonly used (Robertson et al., 2024).

**Figure 7:**
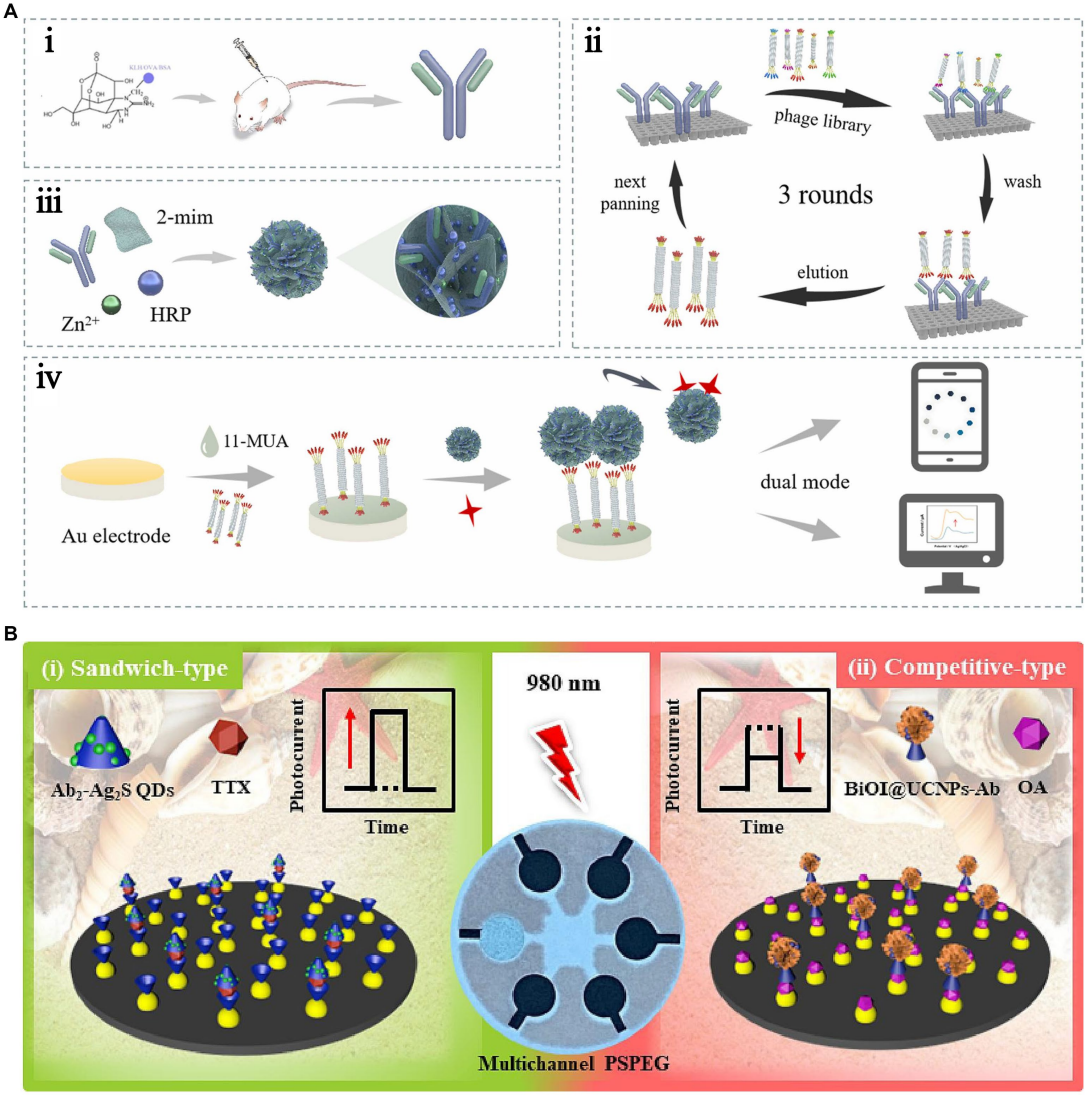
Immunosensors using antibodies as recognition elements. **(A)** Schematic illustration of the electrochemical and colorimetric dual-mode immunosensor. The illustration has been adapted and modified with permission acquired from Li et al. (2023). **(B)** Schematic illustration of the sandwich-type and competitive-type PEC immunosensor. The illustration has been adapted and modified with permission acquired from Zheng et al. (2024).

Kreuzer et al. (2002) were the first to use alkaline phosphatase (ALP) to label anti-TTX antibodies. They modified the TTX–BSA complex on a screen-printed electrode to construct an electrochemical immunosensor. TTX-BSA directly competes with free TTX in the reaction system to bind with ALP-labeled antibodies. The LOD was 0.016 ng/mL, the detection range was 0.1–100 ng/mL, and the detection time was 35 min. This strategy decreases the detection time and improves the detection sensitivity. However, the limitations due to the stability and repeatability of the coupled complex should be addressed. Neagu et al. (2006) first used ALP to label the TTX antigen and modified it on a screen-printed electrode. The electrochemical immunosensor was constructed using differential pulse voltammetry and a direct competitive reaction via ELISA. The LOD was 1 ng/mL, and the detection range was 2–50 ng/mL. Because this electrochemical immunosensor should fix the TTX-coupling complex on electrodes, the complex can hinder the electron transfer process; thus, decreasing the sensing sensitivity. Similarly, Reverté et al. (2017b) also faced the same issue. TTX was immobilized on the gold electrode array, and the LOD was 2.6 ng/mL. To further address the limitations due to low sensitivity and potential harm to operators, a nontoxic and electrochemical and colorimetric dual-mode immunosensor was constructed using an anti-TTX monoclonal antibody (mAb) and phage-displayed mimotope with specific binding to the mAb (Li et al., 2023). A biomimetic mineralized material encapsulated with mAb and horseradish peroxidase (HRP) (HRP/anti-TTX mAb@ZIF-8) was used as the recognition element and signal probe (Figure 7A). This dual-mode immunosensor helps in achieving the simultaneous detection of TTX in seafood samples. The simultaneous detection of multiple marine toxins is crucial for seafood safety. Thus, a stable reading platform was developed by using an electrochemical workstation and a portable reading platform with a smartphone mini program, which was convenient for users to choose according to practical requirements. Zheng et al. (2024) established competitive-type and sandwich-type near-infrared (NIR) light-responsive photoelectrochemical (PEC) multichannel immunosensors for OA and TTX, respectively, with LODs of 5 and 7 pg./mL (Figure 7B). The portable PEC immunosensors were successfully used to detect *Nassariidae* samples simultaneously. These immunosensors allow on-site detection; however, they do not simplify the pretreatment of samples.

**Figure 8:**
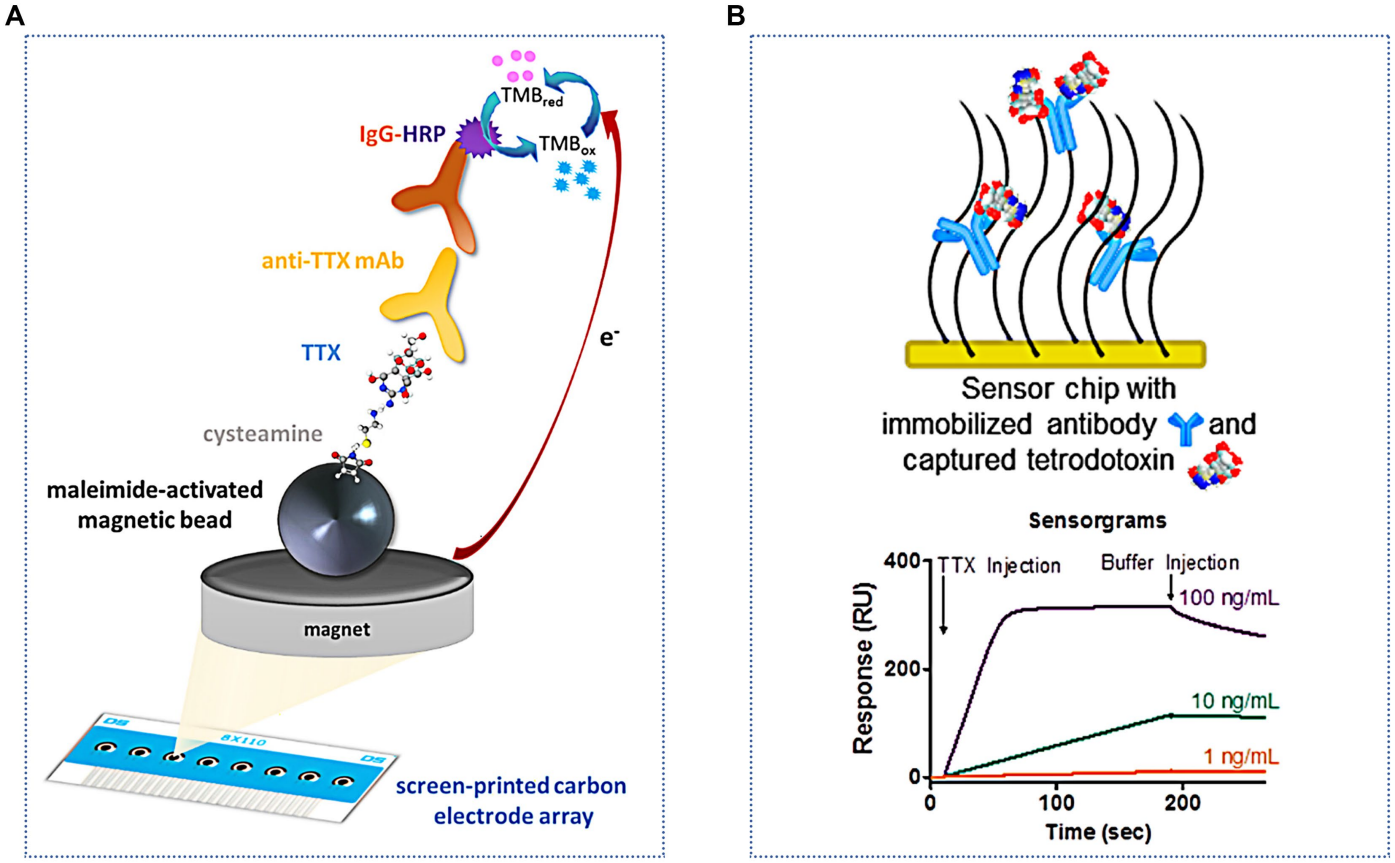
Immunosensors for indirect and direct TTX analysis. **(A)** Schematic illustration of the indirect electrochemical immunosensor based on MB. The illustration has been adapted and modified with permission acquired from Leonardo et al. (2019). **(B)** Schematic illustration of the SPR immunosensor used for direct analysis. The illustration has been adapted and modified with permission acquired from Yakes et al. (2014).

Incorporating magnetic nanoparticles into the sensor constitutes a pivotal strategy for augmenting sensitivity, achieved via the efficacious capture and preconcentration of target compounds. Simultaneously, this approach facilitates rapid separation of analytes by harnessing the capabilities of an external magnetic field, thereby enhancing both the efficacy and speed of the sensing process. Of these, recent studies have shown that using magnetic beads (MB) as a TTX immobilization material can aid in sample pretreatment and improve detection (Zhang et al., 2016). As shown in Figure 8A, this method can decrease the matrix effect, increase the immune response, and simplify washing steps. This method was successfully applied for the rapid screening of TTX in two juvenile pufferfish, *Lagocephalus sceleratus*, captured from the northern Aegean Sea (Greece), with a low LOD of 1.2 ng/mL (Leonardo et al., 2019). Electrochemical immunosensors can fulfill the sensitivity requirements of TTX detection, and they take less time. However, there is still much scope for development in practical applications. Shang et al. (2017) constructed an electrochemiluminescence immunosensor for the ultrasensitive detection of TTX. This sensor used a graphene/Fe_3_O_4_-Au magnetic capture probe and luminol-modified AuNPs to form a double antibody sandwich complex on a glassy carbon electrode. The LOD and linear range were 0.01 ng/mL and 0.01–100 ng/mL, respectively. Multifunctional nanocomposites exhibit good water solubility, excellent electron transfer, high stability, and strong luminescence. Compared with electrochemical biosensors, the signal amplification effect of electrochemiluminescence biosensors is more distinct and has higher detection sensitivity. However, this method is greatly influenced by the instrument and the environment; hence, the sensing conditions are more stringent.

Optical immunosensors are also increasingly used to detect TTX. Taylor et al. (2008) modified TTX on a gold film by optimizing a chemical fixation method. This improved the specific reaction of TTX with antibodies and decreased nonspecific binding. An SPR immunosensor method was established, with an LOD of 0.3 ng/mL and a good linear response in the range of 0.01–10,000 ng/mL. SPR immunosensors are useful for testing TTX in different samples, including pufferfish (Vaisocherová et al., 2011), gastropods (Campbell et al., 2013), milk and apple juice (Yakes et al., 2010), and urine (Taylor et al., 2011). In addition to these traditional indirect measurement methods, direct analysis can be achieved using an SPR immunosensor (Yakes et al., 2014) (Figure 8B). The LOD is lower (0.09 ng/mL), the analysis time is shorter, and fewer reagents are needed; therefore, this method has good application in the pufferfish matrix. Most SPR assays need to introduce nanomaterials, such as gold- and silver-based nanomaterials, as labels to enhance the signal response.

Other optical immunosensors have also been developed. Reverté et al. (2017a) developed a portable planar waveguide fluorescence immunosensor, providing a multifunctional device containing nanoarrays. The test sample could be analyzed within 10 min by indirect competition, and the LOD was 2.5 ng/mL. This method can detect 0.4–3.29 μg/g TTX in a pufferfish matrix. Furthermore, the recovery rate was between 85–115%. The optimization of an immunosensor is based on organic light-emitting diode (OLED) technology (Daniso et al., 2024). The assay can detect TTX in mussel samples contaminated at the EFSA-recommended threshold of 44 ng/g and can validate the efficiency of the detection of the biological components. This strategy achieved the miniaturization of the integrated fluorescence reader system and can be used in hand-held portable point-of-care sensing systems. Campàs et al. (2020) quantitatively analyzed TTX contents in Pacific oyster, razor clam, and mussel samples using an MB-based colorimetric immunosensor. The detection time of this method is less than 1.5 h, which is still longer than that of the aforementioned immunosensor.

Recently, metal–organic framework (MOF) nanomaterials, with their large surface area, ultrahigh porosity, tunable pores, and ease of functionalization, are ideal candidates for stabilizing matrices to embed nanoparticles. The resulting hybrid nanomaterials boast exceptional physicochemical properties, underlining their potential applications. For example, oriented antibody (Ab)-decorated and fluorescent quantum dot (QD)-loaded MOF biocomposites (QD@MOF*Ab) were synthesized and endowed with targeting specificity, fluorescence output, and high stability. Thus, a smartphone-based portable fluorescent biosensor was proposed for TTX determination by using cotton swabs and QD@MOF*Ab (Liu et al., 2024b). This immunosensor has a remarkable analysis speed (15 min), which is one to 2 h faster than that of conventional immunoassays. It can be used to determine TTX contents in puffer fish and clam samples and provides a valuable reference for developing portable biosensing platforms for on-site rapid TTX analysis in seafood.

#### 7.4.3 Aptasensor

Aptasensors use aptamers, which are single-stranded oligonucleotides of approximately 40–100 nt, as recognition elements. These aptamers are artificially screened from single-stranded DNA (ssDNA) or RNA libraries and can bind to target molecules with high affinity and specificity. Compared with cells and antibodies, aptamers are easily produced in large quantities, simple to synthesize and modify, possess short synthesis cycles, exhibit low costs, maintain minimal differences between batches, and exhibit a wide range of application targets. The affinity and specificity of these antibodies are comparable to those of other antibodies. Hence, aptamers are referred to as “chemical antibodies.” Their characteristics are summarized in Table 1.

**Table 1:** Comparison of cell, antibody, and aptamer characteristics.

Characteristics	Cell	Antibody	Aptamer	References
Screening method	Cell culture *in vitro*	Animal immunization	Screening *in vitro*	Reverte et al. (2023)
Transport requirement	High	High	Low	Zhang et al. (2023)
Storage period	Short	Short	Long	Dillon et al. (2021)
Stability	Low	Low	High	Crivianu-Gaita and Thompson (2016)
Modification	Difficult	Difficult	Easy	Liu et al. (2020b)
Economic cost	High	High	Low	Liu et al. (2020a)
Sensitivity	Medium (LODs: 0.3–89 ng/mL)	High (LODs: 5 × 10^−3^–2.6 ng/mL)	High (LODs: 2.65 × 10^−4^–1.21 ng/mL)	Goud et al. (2018)
Specificity	Low	High	High	Zhang et al. (2018)
Repeatability	Low	Low	High	Huang et al. (2021)

Researchers have used ligand exponential enrichment system evolution technology (SELEX) to screen specific aptamer sequences for various marine biotoxins. However, screening aptamers for low-molecular-weight compounds, such as TTX, is challenging. Nevertheless, previous studies have screened three aptamers of TTX.

**Figure 9:**
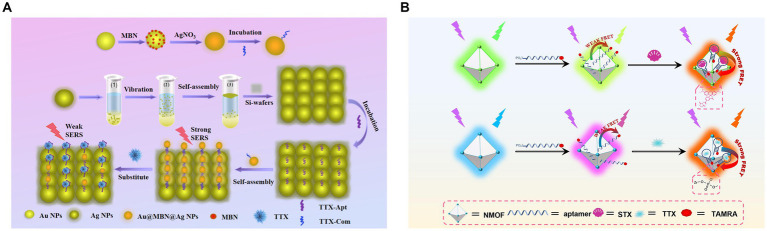
Aptasensors using the first TTX aptamer. **(A)** Schematic illustration of the dual-molecule calibration SERS aptasensor. The illustration has been adapted and modified with permission acquired from Yin et al. (2022). **(B)** Schematic illustration of fluorescent MOF-based aptasensors for STX and TTX. The illustration has been adapted and modified with permission acquired from Dou et al. (2023).

The first TTX aptamer was obtained by SELEX in 2012. Detailed information on the screening process or aptamer affinity has not been provided (Shao et al., 2012). Fomo et al. (2015) developed an electrochemical impedance method using this aptamer, with an LOD of 0.199 ng/mL and a linear range of 0.23–1.07 ng/mL. However, this method did not determine the selectivity toward other toxins, and real samples were not evaluated. Subsequently, this aptamer was also used to construct a berberine-based fluorescence biosensor (Lan et al., 2019), with an LOD of 0.074 nM (approximately 0.024 ng/mL) and a detection range of 0.1–500 nM. This aptasensor possesses the advantages of high specificity, good sensitivity, and rapidity. It can be used for the analysis of spiked serum samples, with a recovery rate of 96.54–106.40%. Therefore, exonuclease I (Lan et al., 2020) was added to this system to remove background fluorescence, which significantly improved the detection sensitivity (LOD of 11 pM, approximately 4 pg./mL) and increased the linear detection range (0.03–6,000 nM). Therefore, this method can be successfully applied to detect pufferfish samples. Yin et al. (2022) constructed a novel bimolecular-calibrated aptasensor based on sensitive surface-enhanced Raman spectroscopy (SERS) (Figure 9A). A silver nanoparticle (Ag NP) monolayer was assembled on a silicon (Si) substrate, and its surface was modified using TTX aptamers. The Raman peak of the Si molecule at 520 cm^−1^ was considered as an internal standard. The complementary chainS of the aptamer and 4-mercaptobenzonitrile (MBN) molecule-functionalized Au@MBN@Ag NP materials were used as detection probes. The Raman intensity ratio of MBN to Si was used to precisely detect TTX. The linear range was 0.0319–319.27 ng/mL, and the LOD was 0.024 ng/mL. The recovery rate of spiked pufferfish was 95.75–99%. Two zirconium fluorescence metal–organic frameworks (MOFs) possessing two fluorescent emissions were constructed by combining TAMRA-labeled aptamers for STX and TTX (Dou et al., 2023) (Figure 9B). The LODs were 1.17 nM and 3.07 nM, respectively. Aptasensors were successfully applied to shellfish samples for toxin detection.

**Figure 10:**
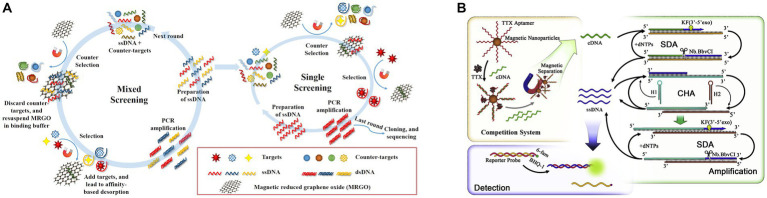
Aptasensors using the second TTX aptamer. **(A)** Schematic illustration of multiple SELEX using MRGO-assisted separation for TTX. The illustration has been adapted and modified with permission acquired from Gu et al. (2018). **(B)** Schematic illustration of the ultrasensitive fluorescent aptasensor based on triple cycle amplification. The illustration has been adapted and modified with permission acquired from Zhang et al. (2020).

Gu et al. (2018) screened another TTX aptamer using magnetic reduced graphene oxide (MRGO) to adsorb ssDNA and for magnetic separation, establishing a non-immobilized multiple SELEX technology based on magnetic separation (Figure 10A). In the same study, aptamers for STX, DA, and TTX were screened, and the TTX aptamer exhibited a dissociation constant (*K*_D_) of 44.12 nM and did not react with other toxins, indicating high affinity and specificity. The fluorescence method showed that the LOD of TTX was 1.21 ng/mL, the detection range was 5–150 ng/mL, and the recovery rate of spiked clam samples was 83.01–98.69%. Subsequently, Zhang et al. (2020) developed an ultrasensitive fluorescent aptasensor using this aptamer (Figure 10B). The aptamer was immobilized on magnetic beads for subsequent competitive binding to TTX and complementary DNA (cDNA) to the aptamer. After the competition, the remaining cDNA was separated by magnetic beads as primers, triggering triple-cycle amplification to obtain more ssDNA. This amplified ssDNA was combined with the reporter probe to achieve TTX fluorescence detection. The aptasensor, combining strand displacement amplification (SDA) and catalytic hairpin assembly (CHA), showed a low LOD of 0.265 pg./mL and exhibited no cross-reactivity with other marine toxins. The recovery rate of spiked shellfish samples was 99.67–116.67%, showing high sensitivity, specificity, and reliability. However, it is important to note that the reaction system is highly complex, and involves many reagents and time-consuming steps. Therefore, it may not be conducive for rapid on-site detection of TTX.

**Figure 11:**
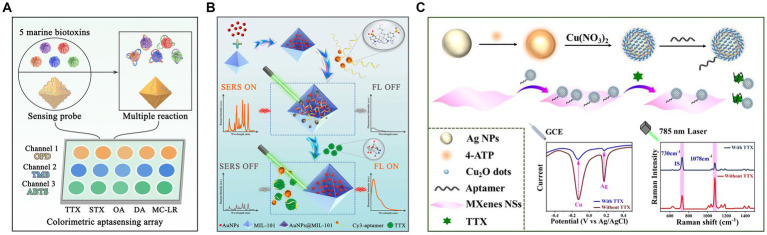
Nanomaterial-based TTX aptasensors. **(A)** Schematic illustration of the discrimination of five marine biotoxins using a colorimetric aptasensing array with high-activity ZrFe-MOF. The illustration has been adapted and modified with permission acquired from Liu S. et al. (2023). **(B)** Schematic illustration of fluorescence/SERS dual-mode aptasensors based on AuNPs@MIL-101. The illustration has been adapted and modified with permission acquired from Liu et al. (2022). **(C)** Schematic illustration of Ag@Cu_2_O NP-engineered electrochemical/SERS dual-mode aptasensors. The illustration has been adapted and modified with permission acquired from Yao et al. (2023).

The rational design of nanomaterials holds immense significance for establishing aptasensors that allow the quantitative and accurate detection of TTX in complex systems. Liu S. et al. (2023) used Fe(III) and Zr(IV) to prepare bimetal–organic frameworks (ZrFe-MOF) with peroxidase-like activity, which was inhibited by aptamers. This colorimetric aptasensor achieved an LOD of 0.07 ng/mL and a detection range of 0.1–200 ng/mL. Furthermore, this method could successfully differentiate multiple marine biotoxins in seawater via a pattern recognition process (Figure 11A). Similarly, a label-free ratiometric fluorescent aptasensor was developed using the multifunctional ZrFe-MOF (Liu et al., 2024a). These aptasensors primarily rely on a single-sensing mode, which may be susceptible to variations in assay environments, apparatus, and operations. To address this limitation, an ultrasensitive fluorescence and SERS dual-mode aptasensor was used with AuNP-embedded MOF nanohybrids (AuNPs@MIL-101) (Figure 11B). Their detection sensitivities were 6 and 8 pg./mL, respectively. Furthermore, the two quantitative detection approaches could achieve satisfactory spiked recoveries in pufferfish and clam samples (Liu et al., 2022). Using dual-signal responses, electroactive and SERS-active Ag@Cu_2_O NPs were fabricated, modified by TTX aptamers, and assembled using MXene nanosheets (NSs) (Figure 11C) due to their large surface area, good conductivity, and inherent Raman properties (Yao et al., 2023). An electrochemical and SERS dual-mode aptasensor was suggested to have LODs of 31.6 and 38.3 pg./mL, respectively. Multichannel biosensing strategies have improved detection accuracy and reliability, exhibiting great potential for practical applications in quantitative analysis.

**Figure 12:**
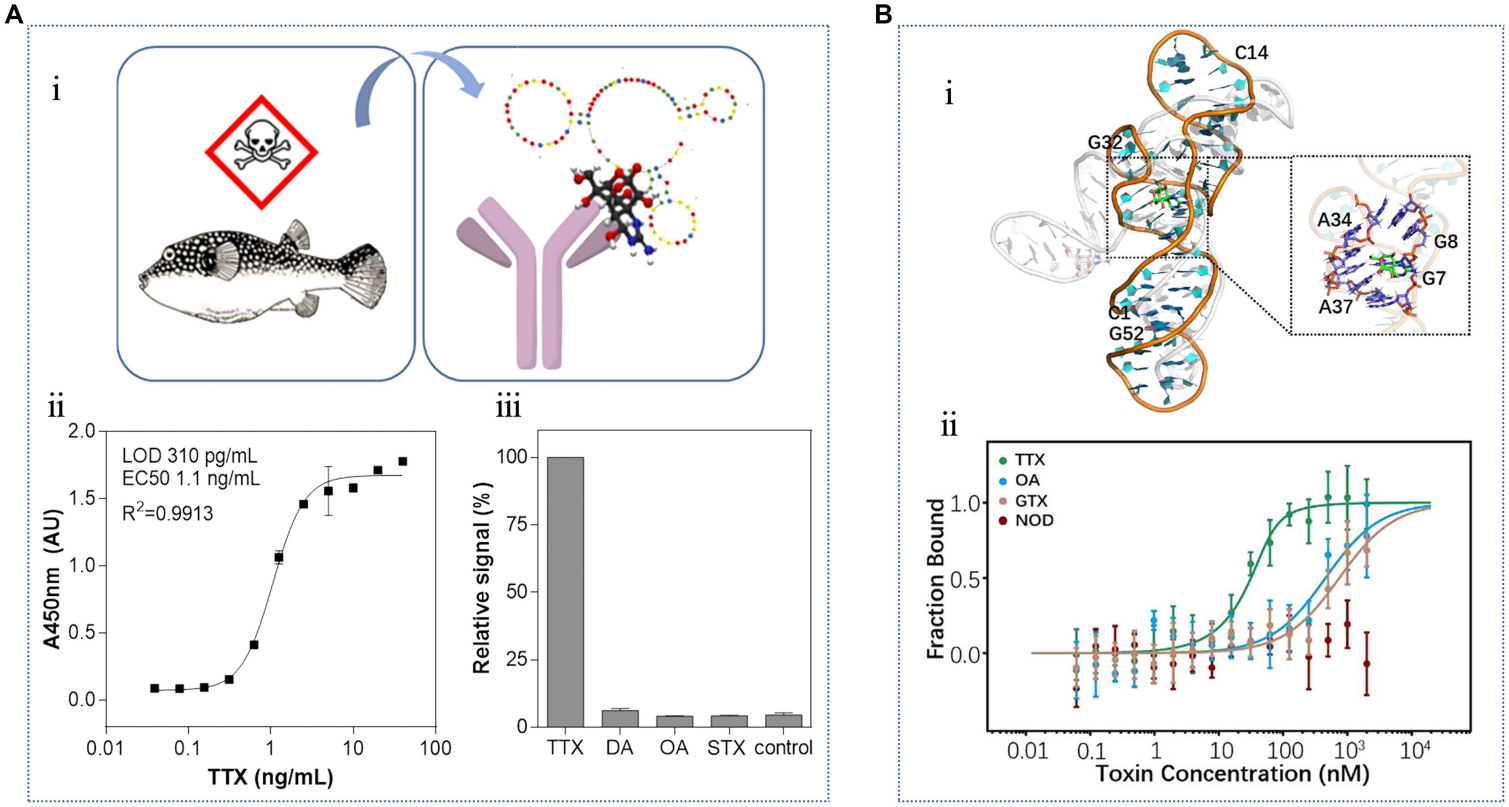
Aptasensors using the third TTX aptamer. **(A)** Hybrid biosensing of antibodies and aptamers: (i) schematic diagram of TTX detection in pufferfish by the sandwich biosensor, (ii) standard curve, and (iii) specificity analysis. The illustration has been adapted and modified with permission acquired from Shkembi et al. (2021). **(B)** Repurposing of thermally stable aptamers: (i) molecular docking of TTX to the aptamer, (ii) binding specificity. The illustration has been adapted and modified with permission acquired from Li et al. (2022).

Recently, Shkembi et al. (2021) used a combination of SELEX and next-generation sequencing technology to screen a new TTX aptamer. As shown in Figure 12A, the oligonucleotide library was immobilized on magnetic beads and used in a mixed antibody-aptamer “sandwich” detection system, which exhibited a *K*_D_ value of 7 nM. This hybrid sensing of antibodies and aptamers combine the advantages of two biorecognition molecules, resulting in excellent sensitivity and specificity. The LOD was 310 pg./mL, and the detection range was between 3.9 × 10^1^–4 × 10^4^ pg./mL. Notably, no cross-reaction was found with DA, OA, or STX. This method was successfully applied to the quantitative analysis of TTX in various tissues of pufferfish, yielding results consistent with those obtained from competitive magnetic bead-based ELISA. Subsequently, Li et al. (2022) used a “repurposing old aptamers for new uses” strategy to develop thermally stable aptamers with nanomolar affinities for TTX (Figure 12B).

Aptasensors can be integrated with electrochemical, fluorescence, SERS, colorimetric, and other sensing methods, and exhibit wide applicability, high stability, and repeatability. However, in terms of sensitivity, these methods may benefit from the addition of nanomaterials or advanced assays to further improve detection performance and expand new functions for widespread applications.

## 8 Comparison among analytical methods

Given complex nature of food matrices and low concentrations of TTX, sample preparation is not trivial and need to be carefully considered during the development of new assays as well as in assessment of their applicability. Typically, the pretreatment strategies for TTX are divided into two primary components: an extraction methodology (extracting target analytes) and a purification procedure (removing interfering substances). Notably, advancements in technology have led to the emergence of techniques that concurrently accomplish both extraction and purification, thereby enhancing efficiency (Long et al., 2020). Furthermore, innovative approaches have been devised that integrate extraction, purification, and preconcentration into a single step, further optimizing the process and reducing overall complexity (Hu et al., 2022). These integrated methodologies offer promising avenues for enhancing the reliability and reproducibility of TTX analysis. A comprehensive comparison and summation of pretreatment and analytical methodologies for TTX determination have been tabulated in Table 2.

**Table 2:** Summary of TTX pretreatment and analysis techniques.

Analytical techniques	Detection system	Sample matrix	Sample pretreatment	LOD	Recovery	Portability	References
ELISA	Indirect competitive ELISA and colloidal gold immunoassay	Pufferfish	Extraction with acetic acid	4.44 ng/mL; 20 ng/mL	70.93–99.99%	No	Ling et al. (2015)
	Self-assembled monolayer-based immunoassay	Pufferfish	Extraction with acetic acid, and liquid–liquid extraction	230 ng/g	N.D.	No	Reverté et al. (2015)
	Self-assembled monolayer-based immunoassay	Oyster and mussel	Extraction with acetic acid, and solid-phase extraction	19–47 ng/g	29–166%	No	Reverté et al. (2018)
LFA	Colloidal gold test strip	Crucian and clam	Extraction with acetic acid, and homogenization	10 ng/g	74.8–105%	Yes	Li et al. (2020)
	Membrane-based fluorescence quenching immunochromatographic sensor	Standards and pufferfish	Homogenization, incubation	0.78 ng/mL	61.3–70.4%	Yes	Shen et al. (2017b)
	Lateral-flow immunochromatographic strip combined with QDNBs and AuNFs	Pufferfish	Homogenization	0.2 ng/mL	85.5–119.7%	Yes	Shen et al. (2017a)
LC–MS and LC–MS/MS	HILIC	Pufferfish	Solid-phase extraction	2.3 ng/g	N.D.	No	Chen et al. (2017)
	HPLC-MS/MS	Fresh pufferfish samples and heat-processed aquatic products	Ultrasonic-assisted extraction, and solid-phase extraction	0.2 ng/g	90.5–107.2%	No	Ye et al. (2022)
	Porous graphite carbon column	Mussel, clam, scallop, and oyster	Extraction with hydrochloric acid, protein precipitation, and solid-phase extraction	6.245–10.841 ng/g	70–120%	No	Rey et al. (2018)
	HILIC	Scallop and short-necked clam	Extraction with acetic acid, and ion-pair solid-phase extraction	27.4–27.9 ng/g	75.7–96.2%	No	Ochi (2021)
	QuEChERS with LC/Q-Orbitrap-HR-MS	Human serum	Extraction with acetic acid	0.67–2.61 ng/mL	85.3–118.2%	No	Zheng et al. (2023)
Cell biosensor	Neuro-2a cell-based APC system	Pufferfish	Extraction with acetic acid	0.05 mg TTX equiv./kg	N.D.	No	Campàs et al. (2024)
Immunosensor	Immunosensor array platforms based on self-assembled dithiols	Pufferfish	Extraction with acetic acid, and liquid–liquid extraction	2.6 ng/mL	N.D.	No	Reverté et al. (2017b)
	Dual-mode immunosensor with HRP/anti-TTX mAb@ZIF-8	Puffer and *Nassariidae* samples	Extraction with methanol	1.0 × 10^−5^ μg/mL; 1.83 × 10^−4^ μg/mL	89.5–109.5%; 81.9–117.5%	Yes	Li et al. (2023)
	Competitive-type and sandwich-type NIR light-responsive PEC immunosensors	*Nassariidae* samples	Extraction with methanol	7 pg./mL	96.6–124.0%	Yes	Zheng et al. (2024)
	Amperometric immunosensor based on single-walled carbon nanotubes and ionic liquid n-octylpyridinum afluorophosphate	Pufferfish	Homogenization, incubation	5 ng/mL	98.16%	No	Zhang et al. (2016)
	Electrochemical magnetic bead-based immunosensor	Juvenile pufferfish	Extraction with acetic acid	1.2 ng/mL	N.D.	Yes	Leonardo et al. (2019)
	Electrochemiluminescence immunosensor based on graphene/Fe_3_O_4_-Au composites and luminol-modified AuNPs	Muscle	N.D.	0.01 ng/mL	96.0–107.3%	No	Shang et al. (2017)
	SPR immunosensor	Sea snail	Extraction with sodium acetate buffer	N.D.	98–112%	No	Campbell et al. (2013)
	Direct SPR immunosensor	Pufferfish	Dilution	0.091 ng/mL	N.D.	No	Yakes et al. (2014)
	Planar waveguide fluorescence immunosensor	Pufferfish	Extraction with sodium acetate buffer	80 ng/g	85–115%	Yes	Reverté et al. (2017a)
	Immunosensor based on OLED technology	Mussels	Extraction with sodium acetate buffer	44 ng/g	Approximately 100%	Yes	Daniso et al. (2024)
	Magnetic bead-based colorimetric immunoassay	Pacific oyster, razor clam, and mussel	Extraction with acetic acid	N.D.	1–3.3 ng/g	Yes	Campàs et al. (2020)
	Smartphone-based fluorescent biosensor based on cotton swabs and QD@MOF*Ab	Pufferfish and clam	Extraction with acetic acid methanol solution	0.13 ng/mL	92.37–108.94%	Yes	Liu et al. (2024b)
Aptasensor	Exonuclease I-assisted fluorescence aptasensor	Pufferfish	Incubation, liquid–liquid extraction	11 pM	99.63–105.51%	No	Lan et al. (2020)
	Self-assembled and dual-molecule calibration aptasensor	Pufferfish	Distribution in citrate buffer solution	0.024 ng/mL	95.75–99%	No	Yin et al. (2022)
	Two zirconium fluorescence MOFs with TAMRA-labeled aptamers	Shellfish	Extraction with formic acid	3.07 nM	84.79–100.73%	No	Dou et al. (2023)
	Non-immobilized multiple SELEX technology based on magnetic separation	Clam samples	Extraction with acetic acid	1.21 ng/mL	83.01–98.69%	No	Gu et al. (2018)
	Magnetic bead-aptamer competition SDA and CHA system	Clams and shellfish	Homogenization, incubation	0.265 pg./mL	99.67–116.67%	No	Zhang et al. (2020)
	Colorimetric aptasensor based on ZrFe-MOF with peroxidase-like activity	Pufferfish and clam	Extraction with acetic acid and methanol solution	0.07 ng/mL	N.D.	No	Liu S. et al. (2023)
	Ratiometric fluorescent aptasensor based on multifunctional ZrFe-MOF	Pufferfish and clam	Extraction with acetic acid and methanol solution	0.027 ng/mL	93.74–107.53%	No	Liu et al. (2024a)
	Fluorescence and SERS dual-mode aptasensor	Pufferfish and clam	Extraction with acetic acid and methanol solution	6 pg./mL	96.2–105.94%	No	Liu et al. (2022)
	Electrochemical and SERS dual-mode aptasensor based on Ag@Cu_2_O NPs	Fish samples	Extraction with acetic acid	31.6 pg./mL; 38.3 pg./mL	98.0 ± 2.2% − 99.1 ± 1.4%; 97.8 ± 2.4% − 98.8 ± 1.1%	No	Yao et al. (2023)
	Hybrid antibody-aptamer assay	Pufferfish and silver cheeked toadfish	Extraction with acetic acid	0.310 ng/mL	93.5–109.1%	No	Shkembi et al. (2021)

N.D.: not discovered.

LC–MS and LC–MS/MS have gained wider application owing to capability of selective characterization and accurate quantification of TTX and its analogues. However, they need tedious sample processing and extraction to obtain high analysis performance. Recently, immunoassays, immunosensors, and aptasensors have made great progress. Unlike LC–MS and LC–MS/MS, these methods possess simplicity, requiring neither advanced instrumentation nor overly complex pretreatment protocols, while offering exceptional portability. This makes them ideal candidates for on-site and real-time testing, particularly in emergency situations where prompt analysis is crucial.

Among immunoassays, the pursuit of an efficient LFA stands as a vibrant and promising area of research, owing to its unparalleled user-friendliness as a paper-based platform that necessitates minimal volumes of both samples and reagents. To further elevate the sensitivity of LFA, it is highly advisable to delve into the exploration of novel nanomaterials, which hold immense potential in enhancing detection capabilities. Additionally, the integration of LFA with ubiquitous smartphones presents a transformative opportunity, enabling on-site, real-time analysis that could revolutionize diagnostic workflows and facilitate rapid decision-making. Such an innovative approach not only streamlines the analytical process, but also broadens the accessibility and applicability of LFA in diverse fields.

As for immunosensors, electrochemical and electrochemiluminescence immunosensors have garnered significant attention due to their robust development, fueled by the integration of novel functional nanomaterials. This integration has unlocked avenues for substantial sensitivity enhancements. Consequently, future research avenues should prioritize the refinement of working electrodes, ensuring robust immobilization of bio-recognition elements on electrode surfaces, and exploring strategies to amplify electrochemical responses. SPR immunosensors also stand out for their simplicity, specificity, versatility, and label-free assay capabilities. As the field progresses, the development of novel SPR chips and the miniaturization of associated instrumentation represent promising trends that will further advance the capabilities of these immunosensors.

Aptamers, emerging as innovative recognition moieties, offer a promising avenue for the advancement of biosensors with enhanced performance. Aptasensors are regarded as superior alternatives to traditional antibody-based immunosensors in rapid and efficient detection paradigms, owing to their cost-effectiveness, swift response kinetics, unparalleled specificity, and remarkable sensitivity. To further elevate detection sensitivity, the exploration of nanomaterials-integrated strategies for signal amplification holds great potential. Notably, the pursuit of aptasensors driven by multiple mechanisms is poised to become a burgeoning research frontier, as it inherently fosters more reliable and precise quantification of target analytes. At last, Table 3 succinctly encapsulates the merits and limitations of the proposed methodologies, providing a comprehensive overview for future directions and refinements.

**Table 3:** Comparison of the characteristics of bioassays, immunoassays, instrumental analysis, and biosensors for TTX detection.

Methods	Classification	Advantages	Limitations	Application	References
Bioassay	MBA	Intuitive results; no need for precision instruments	Poor accuracy and repeatability; time consumption and exertion for animal feeding; ethical issues; unable to distinguish analogs	Qualitative or semiquantitative analysis; animal toxicology study	Suzuki (2016)
	Cell bioassay	No ethical issues; simpler and more sensitive than MBA	Long time for cell culture; high requirements for experimental environment; susceptible to subjective judgment; unable to distinguish analogs	Qualitative or semiquantitative analysis; cell toxicology study	Guzmán et al. (2007)
Immunoassay	ELISA	No need for precision instruments; simple operation; no need for professionals	Low sensitivity; poor stability and repeatability; high price and short storage time for antibody	Semiquantitative analysis; early screening of large samples	Magarlamov et al. (2017)
	LFA	No need for precision instruments; easy operation; fast detection; compactness and portability	Low sensitivity; poor stability and repeatability; high price and short storage time for antibody	Semiquantitative analysis; early screening of large samples; on-site rapid detection	Ling et al. (2019)
Instrumental analysis	LC-FLD	More sensitive than immunoassay	Derivatization; more cumbersome operation; higher requirements for sample matrices; unable to distinguish analogs	Less application at present	Yotsu-Yamashita et al. (2012)
	GC–MS	More sensitive than immunoassay	Derivatization; more cumbersome operation; time consumption; unable to distinguish analogs	Less application at present	Shoji et al. (2001)
	LC–MS and LC–MS/MS	High sensitivity and accuracy; accurate discrimination of analogs; authoritative results	Need for precision instruments and professionals; high testing costs	High-throughput and trace analysis; confirmatory test	Chen et al. (2011) and Park et al. (2024)
Biosensor	Cell biosensor	High-throughput, noninvasive, real-time, and dynamic monitoring	Cumbersome operation; long analysis time; low sensitivity; inability to distinguish analogs	Semiquantitative analysis; cell toxicology and pharmacology study	Alkassar et al. (2022)
	Immunosensor	High sensitivity and specificity; simple equipment; quick analysis; portability	Poor reproducibility and stability; complicated process for antibody conjugation; high price and short storage time for antibody	Semiquantitative analysis; on-site rapid detection	Tang et al. (2023)
	Aptasensor	High sensitivity, specificity, repeatability and stability; facile and rapid operation; low cost; flexible and diverse functions	Aptamer screening of small molecule targets is difficult; immobilization strategies still need to be studied	Quantitative analysis; on-site rapid detection	Liu et al. (2022)

## 9 Conclusions and perspectives

Due to concerns about food safety, environmental pollution, and bioterrorism threats, the detection and analysis of TTX have garnered increasing attention. In this review, we discuss the background of the TTX and its analytical detection methods. Conventional bioassays and instrument analysis methods cannot fulfill the requirements of daily monitoring of trace TTX. Biosensors offer advantages such as simplicity, high sensitivity, and rapid analysis. Furthermore, they are user-friendly. Biosensors are TTX detection technologies with immense application potential. However, the present study has several shown some limitations. The analytical performance of TTX biosensors can be improved in the following three directions:

(1) The precision of detection can be further improved by addressing the challenges associated with the small molecular weight of TTX, which makes screening aptamers and synthesizing antibodies challenging. The design and application of aptamers are in the developmental stage, especially considering the complex and diverse components present in the marine environment and seafood. Therefore, the currently used analytical methods may have limitations, such as low target-recognition efficiency and high environmental interference. To overcome these constraints, advanced biological functional elements, namely artificial intelligence-driven cells and intricate DNA nanomachines, can be explored as innovative alternatives to aptamers and antibodies for achieving dynamic and precise TTX detection. To harness the full potential of these advancements, the integration of intelligent software or specialized equipment, capable of meticulously collecting sensor signals and constructing comprehensive datasets for sample identification and detection, is herein advocated as a recommended approach. This approach ensures that a robust foundation is laid for subsequent data analysis. Employing state-of-the-art machine learning algorithms (Masson et al., 2023), recognition models can be trained to swiftly and accurately detect and analyze targets within actual samples.
(2) The sensitivity of detection should be further improved. Aptamers and nanomaterials are easy to modify. They can be combined with a signal amplification system to further improve the sensing sensitivity (Wang et al., 2023a). Aptamers can be integrated with clustered regularly interspaced short palindromic repeats (CRISPR)-associated (Cas) systems and nucleic acid amplification techniques to allow multiple signal amplification. Moreover, aptamers can be used to design self-assembled DNA nanostructures for the construction of biosensors based on DNA logic gates. Notably, employing an array of robust technologies and advanced nanomaterials, including MOFs, hydrogels, nanozymes, quantum dots, and transition metal carbides (MXenes), presents avenues to attain heightened sensitivity in analytical platforms. These innovative nanomaterials offer a promising foundation upon which to build, with the potential for further enhancement through the integration of aptamers, thereby fostering the development of high-performance, multifaceted nanomaterials tailored for diverse signal transduction systems. Moreover, to elevate detection sensitivity even further, the incorporation of sophisticated signal amplification strategies, such as hybridization chain reaction amplification (HCR) and rolling circle amplification (RCA), into aptasensing designs emerges as a compelling direction for exploration. These strategies hold the key to amplifying minute analytical signals, enabling more sensitive detection capabilities. Therefore, the development of novel TTX sensing strategies can contribute to a wider detection range and increased sensitivity, fulfilling diverse analytical requirements.
(3) The portability, operability, and ease of storage of aptasensors can be further improved. Many analytical methods require the completion of detection reactions in the liquid phase using nonportable instruments. Challenges due to the preservation of liquid samples and the inconvenience of carrying analytical instruments can pose difficulties for on-site testing. Therefore, future research can focus on developing portable assays for TTX. Concurrently, harnessing portable transducers like smartphones as detection platforms represents a transformative approach that can significantly facilitate the deployment of aptasensors in various settings. By leveraging ubiquitous smartphone technology, aptasensor development becomes more accessible and user-friendly, broadening their applicability. Combined with 3-dimensional (3D) printing technology (Zub et al., 2022), small, portable colorimetric, fluorescent, and Raman detection equipment can be constructed by optimizing processing techniques. This small device can replace current methods, thereby improving the portability and operability of instruments for field detection. Technologies such as freeze-drying and nano-processing (Lu, 2022) can be used to prepare frozen paper-based chips and flexible sensing substrate membranes, transferring liquid-phase reactions to a solid-phase matrix. Moreover, test kits that can more easily store and transport reagents can be developed. Thus, detection systems have the potential for industrial production.

The leveraging of these formidable tools is intended to usher in a novel era characterized by highly efficient and precise TTX detection, thereby overcoming the limitations of traditional approaches. To achieve these goals, researchers in this field should perform further studies. Some examples mentioned in this review have produced good results. Therefore, future biosensors can produce rich results in applications that efficiently detect trace TTX.
